# *VID22* counteracts G-quadruplex-induced genome instability

**DOI:** 10.1093/nar/gkab1156

**Published:** 2021-12-06

**Authors:** Elena Galati, Maria C Bosio, Daniele Novarina, Matteo Chiara, Giulia M Bernini, Alessandro M Mozzarelli, Maria L García-Rubio, Belén Gómez-González, Andrés Aguilera, Thomas Carzaniga, Marco Todisco, Tommaso Bellini, Giulia M Nava, Gianmaria Frigè, Sarah Sertic, David S Horner, Anastasia Baryshnikova, Caterina Manzari, Anna M D’Erchia, Graziano Pesole, Grant W Brown, Marco Muzi-Falconi, Federico Lazzaro

**Affiliations:** Department of Biosciences, Università degli Studi di Milano, Via Celoria 26, 20133 Milan, Italy; Department of Biosciences, Università degli Studi di Milano, Via Celoria 26, 20133 Milan, Italy; Department of Biosciences, Università degli Studi di Milano, Via Celoria 26, 20133 Milan, Italy; Department of Biosciences, Università degli Studi di Milano, Via Celoria 26, 20133 Milan, Italy; Istituto di Biomembrane, Bioenergetica e Biotecnologie Molecolari, Consiglio Nazionale delle Ricerche, Bari, Italy; Department of Biosciences, Università degli Studi di Milano, Via Celoria 26, 20133 Milan, Italy; Department of Biosciences, Università degli Studi di Milano, Via Celoria 26, 20133 Milan, Italy; Centro Andaluz de Biología Molecular y Medicina Regenerativa-CABIMER, Universidad de Sevilla, Seville, Spain; Centro Andaluz de Biología Molecular y Medicina Regenerativa-CABIMER, Universidad de Sevilla, Seville, Spain; Centro Andaluz de Biología Molecular y Medicina Regenerativa-CABIMER, Universidad de Sevilla, Seville, Spain; Dipartimento di Biotecnologie Mediche e Medicina Traslazionale, Università degli Studi di Milano, via Vanvitelli 32, 20129 Milan, Italy; Dipartimento di Biotecnologie Mediche e Medicina Traslazionale, Università degli Studi di Milano, via Vanvitelli 32, 20129 Milan, Italy; Dipartimento di Biotecnologie Mediche e Medicina Traslazionale, Università degli Studi di Milano, via Vanvitelli 32, 20129 Milan, Italy; Department of Biosciences, Università degli Studi di Milano, Via Celoria 26, 20133 Milan, Italy; Department of Experimental Oncology, IEO, European Institute of Oncology IRCCS, Via Adamello 16, 20139 Milan, Italy; Department of Biosciences, Università degli Studi di Milano, Via Celoria 26, 20133 Milan, Italy; Department of Biosciences, Università degli Studi di Milano, Via Celoria 26, 20133 Milan, Italy; Istituto di Biomembrane, Bioenergetica e Biotecnologie Molecolari, Consiglio Nazionale delle Ricerche, Bari, Italy; Department of Molecular Genetics and Donnelly Centre, University of Toronto, Toronto, Canada; Istituto di Biomembrane, Bioenergetica e Biotecnologie Molecolari, Consiglio Nazionale delle Ricerche, Bari, Italy; Istituto di Biomembrane, Bioenergetica e Biotecnologie Molecolari, Consiglio Nazionale delle Ricerche, Bari, Italy; Dipartimento di Bioscienze, Biotecnologie e Biofarmaceutica, Università di Bari ‘A. Moro’, Bari, Italy; Istituto di Biomembrane, Bioenergetica e Biotecnologie Molecolari, Consiglio Nazionale delle Ricerche, Bari, Italy; Dipartimento di Bioscienze, Biotecnologie e Biofarmaceutica, Università di Bari ‘A. Moro’, Bari, Italy; Department of Biochemistry and Donnelly Centre, University of Toronto, Ontario M5S 3E1, Toronto, Canada; Department of Biosciences, Università degli Studi di Milano, Via Celoria 26, 20133 Milan, Italy; Department of Biosciences, Università degli Studi di Milano, Via Celoria 26, 20133 Milan, Italy

## Abstract

Genome instability is a condition characterized by the accumulation of genetic alterations and is a hallmark of cancer cells. To uncover new genes and cellular pathways affecting endogenous DNA damage and genome integrity, we exploited a Synthetic Genetic Array (SGA)-based screen in yeast. Among the positive genes, we identified *VID22*, reported to be involved in DNA double-strand break repair. *vid22Δ* cells exhibit increased levels of endogenous DNA damage, chronic DNA damage response activation and accumulate DNA aberrations in sequences displaying high probabilities of forming G-quadruplexes (G4-DNA). If not resolved, these DNA secondary structures can block the progression of both DNA and RNA polymerases and correlate with chromosome fragile sites. Vid22 binds to and protects DNA at G4-containing regions both *in vitro* and *in vivo*. Loss of *VID22* causes an increase in gross chromosomal rearrangement (GCR) events dependent on G-quadruplex forming sequences. Moreover, the absence of Vid22 causes defects in the correct maintenance of G4-DNA rich elements, such as telomeres and mtDNA, and hypersensitivity to the G4-stabilizing ligand TMPyP4. We thus propose that Vid22 is directly involved in genome integrity maintenance as a novel regulator of G4 metabolism.

## INTRODUCTION

DNA molecules are intrinsically unstable (1) and are often damaged by exposure to a variety of endogenous and exogenous genotoxic agents (2). When not correctly recognized and repaired, lesions can impede DNA duplication and endanger faithful transmission of the genetic material to the progeny. To preserve genome integrity, eukaryotic cells possess evolutionarily conserved mechanisms that act by handling problems or errors arising during DNA replication, repairing DNA lesions, monitoring chromosome segregation and ensuring proper coordination of all these processes with cell cycle progression (3–7). Failure of any of these DNA integrity pathways leads to a condition known as ‘genomic instability’, characterized by the accumulation of unrepaired DNA damage and genetic alterations ranging from point mutations, insertions/deletions of few nucleotides, or expansion/contraction of repeated sequences, to gross chromosomal rearrangements and aneuploidy (8). Genomic instability impairs cell viability and compelling experimental evidence obtained in recent years in human cells shows that it acts as a driving force during tumorigenesis (9–12).

In addition to genotoxic agents, genome integrity is also threatened by non-canonical nucleic acid secondary structures. Indeed, certain genomic sequences have the potential to form DNA secondary structures such as triplexes, cruciforms, hairpins, DNA:RNA hybrids and G-quadruplexes (G4-DNA) that may interfere with physiological DNA metabolism (13). G4-DNA is one of the most characterized alternatives to the classical double helical DNA conformation and can occur when a stretch of two or more guanines is repeated four times at short distance. Hoogsteen hydrogen bonding between four guanines and monovalent cations induces an extremely stable planar structure called a G-quartet (14,15). Two or more parallel G-quartets form the so called G-quadruplex conformation that can be either intra or intermolecular (involving two or more strands) and each type can display different configurations (16,17).

Single-stranded DNA (ssDNA) formation can spontaneously induce the generation of G-quadruplexes. For this reason, DNA transactions that expose ssDNA, such as replication, transcription and telomere metabolism, promote the folding of G4-DNA (18). If not promptly resolved, these structures can jeopardize DNA replication and can compromise telomere homeostasis. In these contexts, cells have evolved specialized helicases that recognize and unwind G4-DNA to preserve genetic stability. Several G4-helicases have been identified *in vitro* and *in vivo*, including the yeast Sgs1 and Pif1 proteins, and mammalian BLM, WRN, PIF1, and FANCJ (19–23). In addition to potentially detrimental effects, G4-DNA structures may play important regulatory roles. While G4-DNA may be an obstacle for the progression of DNA polymerases, they also appear to be active components of metazoan replication origins (reviewed in (24)). Moreover, about half of all human genes contain sequences prone to G4-DNA formation in their promoter regions (25,26), suggesting a role for these structures in regulating gene expression. In accordance, the promoter regions of KRAS and c-MYC genes can form G4-DNA and, if stabilized, these structures repress transcription (27,28).

The identification and analysis of the factors that contribute to preserving genome integrity is a major source of information for understanding tumorigenesis and defining new potential therapeutic targets. While many molecular mechanisms underlying DNA metabolism are now well understood, several regulatory aspects are yet unclear, hinting at the involvement of still unidentified players. Genetic screens in budding yeast represent a powerful strategy for the identification of such factors. From a Synthetic Genetic Array (SGA) screen to identify new genes required to prevent spontaneous DNA damage, we identified *VID22*, which encodes a non-essential protein that contributes to a heterotrimeric complex with its paralog Env11 and the essential Myb domain telomere-binding protein Tbf1 (29,30). Vid22 is a nuclear protein (31) containing two domains whose functions are not well described, a BED-type zinc finger domain and a RNaseH-like domain (32,33). Previous reports indicate its involvement in double strand break (DSB) repair, and a role with Tbf1 in modulating histone occupancy in proximity of DSB has been suggested (34,35). Moreover, Vid22 chromatin immunoprecipitates are significantly enriched in genes associated with predicted G4 regions (35), suggesting that Vid22 could interact with G4-DNA *in vivo*.

Here, we delineate a role for Vid22 in promoting the stability of G4-DNA regions *in vivo*. Whole genome sequencing of *vid22Δ* mutant strains allowed the characterization of the genomic loci and the types of mutations that accumulate spontaneously in the absence of Vid22. We observed frequent gross chromosomal rearrangements (GCRs) in the proximity of G4-DNA structures as well as alterations in telomeric regions and loss of mitochondrial DNA, all of which are genomic regions that present high densities of G4 motifs. Accordingly, loss of Vid22 increases cell sensitivity to the TMPyP4 (5,10,15,20-tetratkis-(*N*-methyl-4-pyridyl)-21,23-*H*-porphyrin) ligand that binds and stabilizes G4 structures (36). We find that purified Vid22 is able to bind directly DNA G4-forming sequences *invitro*, and that Vid22 is enriched on chromatin at G4-DNA and suppresses chromosomal aberrations at G4-DNA structures. Together, our data indicate that Vid22 controls G4-DNA metabolism to maintain genome integrity.

## MATERIALS AND METHODS

### Yeast strains, growth conditions and plasmids

All the strains and plasmids used in this work are listed in Supplementary Table S1. All the yeast strains were derived from BY4741, BY4742 and BY4743 (37), except strains used for GCR assays that derive from the YPH500 background (38). Yeast strains were obtained by standard procedures of transformation and tetrad dissection. A one-step PCR approach was used to delete genes and to tag proteins at the C-terminus, as described in (39). The eleven unrelated *vid22Δ* strains used in the PFGE analysis were obtained by independent one-step deletions. For the analysis shown in Supplementary Figure S2B, wild-type and *vid22Δ* mutants were obtained by tetrad dissection of diploid YNOV344 (*VID22/vid22Δ*) and grown in YEPD medium in the same conditions so that cells would go through the same number of cell divisions. Plateau-phase cultures were diluted with fresh medium to allow re-growth into plateau phase three times. Finally, cells were plated on YEPD to collect isolated clones for the PFGE analysis.

In the yeast strain YFL2922, the G4 in position Chr VIII 512264–512325 (17) was mutated using the ‘Delitto Perfetto’ strategy (40). Briefly, the pCORE cassette was amplified by PCR using G4_VIII_DPfor (5′-ATA TAA TCA GGG CTT AAG TAA ACG CTT CGC TGT GAT TTC CGA GCT CGT TTT CGA CAC TGG-3′) and G4_VIII_DPrev (5′-ATT TAA GAA AAA CTT TTT TTT TTT TTT TTT CGG ATG ATA TTC CTT ACC ATT AAG TTG ATC-3′) oligos and transformed in YGE651.2. The ‘Delitto Perfetto’ cassette was then replaced by dsDNA containing the desired mutations obtained as described below. First, we amplified genomic DNA fragment 1 by PCR using oligos G4_VIII_Afor (5′-CCT CAT CTA TAT ATA ATC AG-3′) and G4_VIII_Brev (5′-CGC TCA CTT ATG GCA TCG CTA TTA TAG CAA AAC CCT GCT T-3′), and fragment 2 using oligo G4_VIII_Cfor (5′-AAC ACA AGT ATA AGC AGG GTT TTG CTA TAA TAG CGA TGC CAT AAG TGA GCG CAG GGC TCA-3′) and G4_VIII_Drev (5′-TTG ACA AAT GTT TCA GAT CC-3′). Then, the PCR fragments 1 and 2 were annealed and amplified using oligos G4_VIII_Afor and G4_VIII_Drev. The oligos Brev and Cfor contain the desired mutations. A similar strategy was used to mutagenized Tbf1-BS, using the following oligos: DP_VIII_NEW_for (5′-AAG TCG ATT AAA AGT AGG GCT AAC ACA AGT ATA AGC AGG GGA GCT CGT TTT CGA CAC TGG-3′), DP_VIII_NEW_rev (5′-TGA GCC CTG CCC TCA CTT ATG GCA TCC CTA TTA TAG CAA ATC CTT ACC ATT AAG TTG ATC-3′), TBF1_BS_mut_Bfor (5′-AAA GTC GAT TAA AAG CCG GGC TAA CAC AAG TAT AAG CAG GGT TTT GCT ATA ACC GGG ATG-3′), TBF1_BS_mut_Crev (5′-TGA GCC CTG CCC TCA CTT ATG GCA TCC CGG TTA TAG CAA AAC CCT GCT TAT ACT TGT GTT-3), Tbf1_BS_mut_Afor (5′- AAA AAA AAA AAA AAA AAG TTT TTC TTA AAT GCA TAG GTT TAA AGT CGA TTA AAA GTC GGG-3′), TBF1_BS_mut_Drev (5′-AAA AAT TCA GAA TTT CGG AAA ATC CAT GTA CGC GCA TCG ATG AGC CCT GCC CTC ACT TAT-3′), Tbf1_BS_G4_mut_Bfor (5′-AAA GTC GAT TAA AAG TCG GGC TAA CAC AAG TAT AAG CAG GGT TTT GCT ATA ACC GCG ATG-3′), Tbf1_BS_G4_mut_Crev (5′-TGA GCC CTG CGC TCA CTT ATG GCA TCG CGG TTA TAG CAA AAC CCT GCT TAT ACT TGT GTT-3′), Tbf1_BS_mut_Afor (5′-AAA AAA AAA AAA AAA AAG TTT TTC TTA AAT GCA TAG GTT TAA AGT CGA TTA AAA GTC GGG-3′), Tbf1_BS_G4_mut_Drev (5′-AAA AAT TCA GAA TTT CGG AAA ATC CAT GTA CGC GCA TCG ATG AGC CCT GCG CTC ACT TAT-3′).

For all the experiments, cells were grown at 28°C in YEP medium (1% yeast extract, 2% peptone) with 2% glucose (YEPD), with 2% raffinose (YEPR), or with 2% raffinose and 2% galactose (YEPRG). For strains carrying plasmids, cells were grown in Synthetic-Complete (SC) medium (6.7 g/l yeast nitrogen base) containing the appropriate sugar(s) at 2% concentration and nutrients to maintain the selection. For TMPyP4 treatment, overnight cultures grown in SC medium supplemented with 2% glucose were treated with 0–20–30–40 μM of TMPyP4 for 2 h and then plated on YPED. The cell cycle phase was evaluated using flow cytometric analysis using FlowJo v10 software (41). Plasmids pNOV1.4 and pFL82.4 were obtained by inserting the *NAT^R^* marker gene amplified from pAG25 plasmid using NAT1_F (5′-GTC GAC ACA TGG AGG CCC AGA ATA CCC-3′) and NAT1_R (5′-GTC GAC CAG TAT AGC GAC CAG CAT TCA C-3′) oligos, into the SalI digested pRS426 and pFL60 vectors respectively. pFL60 was obtained by cloning *GAL1pr-DDC2* SacI/XhoI fragment obtained by digestion of pML105 (42) into pRS426. Plasmid pFL183.1 was obtained by performing a standard PCR protocol for site-directed mutagenesis using plasmid KP118 as a template (oligoF 5′-CCA AGC GGT AAA ACT TAC ATG CGA TGG TGG CGT CAC ATG GGT GGT CCA AAG-3′; oligoR 5′-CTT TGG ACC ACC CAT GTG ACG CCA CCA TCG CAT GTA AGT TTT ACC GCT TGG-3′). Plasmid pFL178.1 was obtained by cloning Chr VIII from coordinates 512240 to 512755 amplified with ChrVIII_for (5′-CTA GTC TAG AAA TGC ATA GGT TTA AAG TCG-3′) and ChrVIII_rev (5′-CTA GTC TAG ACT ATT TAT GGT GGA AAA GCT C-3′) oligos into XbaI digested pRS415, while pGELE50.1 was obtained by amplifying the G4 mutated DNA version from yeast strain YFL2922 as the template using the same strategy described for the pFL178.1.

pGELE39.1 plasmid was obtained by cloning *VID22* sequence amplified from yeast genomic DNA using Vid22BamHI_F (5'-CGC GGA TCC GAT GAG AGC GAT GGA CAC ACA G-3') and Vid22XhoI_R (5'-AAC CGC TGC AGC TAT GGA AGA TAC TGA CTT GC-3') oligos in BamHI/XhoI digested pRSETb vector.

Plasmid pGELE48 carrying mutation in *VID22 BED* domain C87A-C90R-H110Y-H115Y (32) was obtained by performing a site directed mutagenesis (Quik Change Agilent) in two steps on pGELE39.1 using for step 1 oligos VBED_C87A_C90R_FW (5'-ATG CTG GAG GCA GTA AAA GCC AAG TAC CGC GGT GTG ATA ATA AGA CGG-3') and VBED_C87A_C90R_REV (5'-CCG TCT TAT TAT CAC ACC GCG GTA CTT GGC TTT TAC TGC CTC CAG CAT-3'), and for step 2 oligos VBED_H110Y_H115Y_Fw (5'-GAA GCC TCG CAA ACT TAT TTG TGG AGC ACG TAT AAG ATA GAC CCG A-3') and VBED_H110Y_H115Y_Rev (5'-TCG GGT CTA TCT TAT ACG TGC TCC ACA AAT AAG TTT GCG AGG CTT C-3').

### *DDC2* synthetic dosage lethality screen

The genetic screen was performed using SGA technology (43). Briefly, the query strain carrying the inducible *DDC2*-overexpressing plasmid (pFL82.4) was crossed to an ordered array of all the viable yeast deletion strains. Diploid cells were transferred to a sporulation-inducing medium, after which the germinated spores were selected for the simultaneous presence of the gene deletion and the plasmid. Cells were then transferred to a galactose-containing medium to induce *DDC2* overexpression, and colony size was analyzed as a measure of fitness, as described in (43). The whole procedure was performed in parallel with a control query containing the same vector devoid of the *GAL1pr-DDC2* gene (pNOV1.4). The screening was performed in triplicate and SGA scores and p-values were calculated as indicated in (43). For each replicate, an intermediate cut-off (ϵ < −0.08, *P* < 0.05 (43)) was applied and a total of 52 mutant strains identified in at least two replicates were selected for further analysis. The colony size and SGA score data are presented in Supplementary Table S2.

### Spatial analysis of functional enrichment

Network annotations were made with the Python implementation of spatial analysis of functional enrichment (SAFE) ((44); https://github.com/baryshnikova-lab/safepy). The yeast genetic interaction similarity network and its functional domain annotations were obtained from (45).

### Serial dilution growth tests

Log-phase yeast cultures were diluted to 2 × 10^6^ cells/ml. A series of 10-fold dilutions were prepared and spotted on YEP plates or selective plates containing the appropriate sugar. Plates were incubated at 28°C for 2–3 days. Where indicated, the plates were irradiated with 50 J/m^2^ using a UV Stratalinker 2400 (Stratagene) or 100mM HU (USBiological, cat. H9120) was added.

### Yeast genomic sequencing

Yeast DNA was prepared using MasterPure^TM^ Yeast DNA Purification Kit (Epicentre, MPY80200) and quantified by fluorimetric assay using the Quant-iTTM PicoGreen^®^ dsDNA Kit (Invitrogen, Carlsbad, CA, USA). Libraries were prepared using the Nextera XT library preparation workflow (Illumina, San Diego, CA, USA) to obtain DNA fragments ranging in size from 600 to 1400 bp approximately. Sequencing was carried out on the Illumina MiSeq platform generating about 8 million 250 × 2 paired-end reads for each sample. Raw sequencing data for the 11 *vid22* mutants and one control *S. cerevisiae* BY4741 strain have been deposited in the Short Read Archive (SRA) under Bioproject PRJNA646604. Data can be accessed through the following link: https://www.ncbi.nlm.nih.gov/sra/PRJNA646604.

### SDS-PAGE and western blotting

Protein extracts were prepared in trichloroacetic acid (TCA) as described in (46) and separated by SDS-PAGE. Western blotting was performed with anti-Rad53 (gift from C. Santocanale) or anti-HA (12CA5) as primary antibody, and Goat anti-Mouse HRP (ThermoFisher-Scientific, cat.31430) or Goat anti-Rabbit HRP (ThermoFisher-Scientific, cat.31460) as secondary antibody using standard techniques. Anti-Rad53 signal was detected using film (Amersham Hyperfilm ECL), while anti-HA signal was acquired using a ChemiDoc™ Touch Imaging System (BioRad).

### Chromatin immunoprecipitation (ChIP) assays

Yeast strains used for direct ChIP measurements were obtained by 13Myc tagging of *VID22*. Overnight cell cultures pre-grown in YEP medium containing the appropriate sugar were diluted into fresh medium to a cell density of 4 × 10^6^ cells/ml in 50 ml and grown at 28°C until they reached a density of 1.5–2 × 10^7^ cells/ml before being collected for further analysis. Cells were cross-linked with 1% formaldehyde (Sigma) for 10 min at 25°C. Cross-linking was quenched by addition of 125 mM glycine. After harvesting cells, the pellet was resuspended in NP-40 ChIP lysis buffer (1% NP-40, 140 mM NaCl, 50 mM HEPES (pH 7.5), 1 mM EDTA (pH 8.0), 0,1% sodium deoxycholate, 1 mM phenylmethylsulfonyl fluoride, cocktail proteases inhibitors (Roche)). Yeast whole cell extracts were prepared by FastPrep^®^ FP120 Cell Disrupter (Thermo) in NP-40 ChIP lysis buffer. Following lysate clarification by centrifugation at 16 000 × g for 30 min at 4°C, pellets were resuspended in NP-40 ChIP lysis buffer and samples were sonicated for 15 min, 30 s on, 60 s off, at high power at 4°C with a Bioruptor^®^ Plus sonicator (Diagenode). Immunoprecipitation was carried out with anti-MYC (9E10) mouse monoclonal antibody following incubation with Dynabeads M-280 Sheep Anti-Mouse IgG (Life Technologies). Washing steps and reverse crosslinks were performed as reported in (47). Inputs and IPs were purified using Wizard^®^ SV Gel and PCR Clean-Up System (Promega, A9281); DNA was eluted in 80–100 μl of nuclease-free water. Nucleic acids were quantified with Qubit 4 Fluorometer using Qubit dsDNA HS Assay kit (Invitrogen Q32854), and the quantity obtained was 0.5–1 ng/μl for Inputs and 0.1–0.3 ng/μl for IPs. Samples were directly analyzed by qPCR, then stored at −20°C.

### ChIP-seq analysis

Strains used for ChIP-seq protocol were wild type (no tag) as negative control and Vid22-13Myc tag. ChIP was performed as described above, starting from 150 ml of cultures with a cell density of 1.5–2 × 10^7^ cells/ml. DNA obtained from two independent pooled ChIP experiment was quantified using the Qubit dsDNA High Sensitivity Assay Kit (Invitrogen, Q32851). ChIP-seq libraries were generated starting from 5 ng DNA using an in house protocol (48), quality checked on a Bioanalyzer High-Sensitivity DNA Chip (Agilent) and sequenced on an Illumina HiSeq 2000 at 50 bp read length. Reads were aligned to the reference assembly of the *Saccharomyces cerevisiae* genome using the bowtie (49) program with the following parameters ‘-m 1 –best –strata’ to allow up to 1 mismatch and report only the best scoring alignment. PCR duplicates were removed by means of the rmdup utility from the samtools package (50). Peaks were called by means of the MACS2 program with default parameters. A FDR threshold of 10E-10 was applied (51).

### qPCR analysis

For qPCR analysis of ChIP samples, input and immunoprecipitated (IP) DNA were analyzed using primer pairs producing a 89 bp amplicon for the *HHT2* locus (coordinates Chr XIV 575707–575796) (52) and a 177 bps amplicon for the Chr VIII-G4 at *SKN7* locus (coordinates Chr VIII 512387–512564): Chr VIII_F (5′-ATG AGC AAA ATG TGG TCA GC-3′) Chr VIII_R (5′-ACC CAA ACA AAA GCA GCA AG-3′) and HHT2_CDS_A (5′-TCA ATC TTC TGC TAT CGG TGC TT-3′) HHT2_CDS_B (5′-GCG TGA ATA GCA GCC AGA TTA GT-3′). Oligos for qPCR were purchased from Eurofins Genomics.

PCR reactions were performed in 25 μl total volumes containing 12,5 μl 2× Quantitative Master Mix with SYBR^®^ Green (GeneSpin proprietary formulation ready-to-use containing Xtra Taq Pol, dNTPs, MgCl_2_ and stabilizers optimized for use in real time PCR amplification), primers (200 nM Chr VIII; 130 nM *HHT2*) and template (2 μl 1:100 INPUT or undiluted IP). All components were mixed in 96-well hard-shell PCR plates with clear wells (Bio-Rad), which were sealed with optically clear adhesive Microseal^®^ ‘B’ seals (Bio-Rad) and centrifuged at 1000×*g* for 10 s. The thermal conditions during reaction were 3 min at 95°C followed by 39 thermal cycles at 62°C for 30 s and 95°C for 10 s. All PCR reactions were assembled manually and qPCR experiments performed on a CFX Connect Real-Time PCR System (Bio-Rad). We accepted PCR efficiencies between 95–105%. All PCR reactions were performed in technical triplicates and all biological experiments were performed at least in triplicates. ΔC_T_ (Cycle Threshold) was first calculated, as the difference between IP and input C_T_ values (after correcting for input sample dilution), for both the target gene and the *HHT2* gene (chosen as an internal standard). The target gene enrichment in IP DNA was then calculated using PfaffI method (53). Means of fold enrichment and standard errors of the mean were calculated for biological replicates and unpaired Student's *t*-test was used to determine statistical significance between samples.

### Pulsed field gel electrophoresis

PFGE was performed using the Pulsaphor system with a hexagonal electrode array (Amersham Pharmacia Biotech). Agarose plugs with yeast chromosomal DNAs were prepared as previously described (54). For standard chromosome separation, plugs were loaded in a 1% agarose gel in 0.5× TBE and sealed in the gel using LMP agarose 0.5% in 0.5× TBE. The running conditions were 165 V with 60 s pulses for the first 12 h and 90 s pulses for the last 12 h at 8°C. To visualize DNA, the gel was stained in a solution with 2 μg/ml Ethidium Bromide in 0.5× TBE for 30 min; VersaDoc or Chemidoc (Bio-Rad) were used to acquire images of the stained gels.

### Measurement of telomere length using Southern blotting

Yeast genomic DNAs were extracted using standard methods starting from plateau-phase cultures. DNA samples were digested with XhoI or SalI restriction enzymes; the obtained fragments were separated by electrophoresis in 0.8% agarose 1× TBE. Radiolabeled (DECAprime™ II Kit Invitrogen) specific probes were used to visualize telomeric poly(GT) tails (55) and subtelomeric regions (56). A standard protocol was used for hybridization and detection (57).

### Identification of structural variants and copy number alterations, and GC normalization

Raw reads were subjected to quality trimming using the sliding window operation from the Trimmomatic program (58) (average quality 20, window length 8). Assembly was performed with the SPAdes program using the following set of kmers: 33, 55, 77 and 99. Detection of large indels and structural variants was performed by aligning the final assemblies to the reference sacCer3 genome, (downloaded from http://hgdownload.soe.ucsc.edu/goldenPath/sacCer3/bigZips/) by means of the Mummer4 program (59). Large scale structural rearrangements were identified directly from the alignment by using a custom Perl script. Raw reads were aligned to the reference sacCer3 assembly of the yeast genome using the bowtie2 program. Coverage profiles were computed on sliding genomic windows of 200 bp, overlapped by 100 bp, by using bedtools coverage tool. Variant calling was performed by the means of the varscan2 software (60), with default parameters. The rlm function from the R MASS package (61) was applied to perform GC composition normalization. Scaling factors for GC normalization and estimates of the sensitivity of our CNV detection assay before and after the application of GC content normalization are reported in Supplementary Tables S3 and S4 respectively. Copy Number Variants were identified by pairwise comparisons of coverage profiles of matched genomic windows between the wild-type strain and each *vid22Δ* strain by means of the chi-squared test. The Benjamini-Hochberg correction was applied to control the false discovery rate. A cut-off FDR of 0.05 was used for the identification of genomic windows showing significantly altered coverage. Finally, overlapping windows were merged using bedtools merge, to derive larger genomic intervals. Intervals of more than 500 bp in size, formed by at least three or more windows showing significantly altered coverage were considered as bona fide CNVs. Mitochondrial genome (mtDNA) copy numbers were estimated by comparing the proportion of NGS reads mapped to the nuclear and mitochondrial genome. To avoid confounding factors for the nuclear genome, only single copy *S. cerevisiae* as defined in https://www.nature.com/articles/s41586-018-0030-5 was considered. Coverage levels were normalized by applying the RPKM normalization. Mitochondrial copy number was inferred as the ratio between the average RPKM of single copy nuclear genes and with the mtDNA RPKM.

Intersection of coordinates of genomic features were performed using the bedtools intersect utility (49).

### Gross chromosomal rearrangements

Gross chromosomal rearrangement GCR assays were performed as previously described (62). Different cassettes were integrated into the *PRB1* locus near the *CAN1* gene. GCR rates were measured using the webtool http://flucalc.ase.tufts.edu/ (63) and the MMS maximum likelihood statistical method (64). Briefly, 5 colonies for each strain were grown at 28°C for 3 days to obtain saturated cultures. Cells were plated on SC medium with 5-FOA and canavanine to select GCR events and SC medium to measure the total cell number. The G4 forming sequences tested in the GCR assay are: 5′-**GGG** TCC TCC AAG CGG TAA AAC TTA CAT **GGG** ATG GT**G GGG** TCA CAT **GGG**-3′ (Figure 5B and (21)) and 5′-**GGG** TTT TGC TAT AAT A**GG G**AT GCC ATA AGT GA**G GG**C A**GG G**-3′ (ChrVIII-G4 Figure 6F)

### Reflective phantom interface (RPI) sensor preparation and measurement

Reflective phantom interface (RPI), is an optical label-free biosensor enabling the studying a variety of interactions, including antigen-antibody (65), protein-glycan (66), DNA–DNA (67), RNA–DNA (68) and protein–DNA (69). RPI analysis was employed for the characterization of the interaction between BG4 (70) or Vid22 and different DNA strands immobilized on the RPI sensing surface. The BG4 antibodies were produced with the support of the Protein Purification Facility of the Biosciences Department at University of Milan starting from pSANG10-3F-BG4 plasmid (70). Wild type Vid22 and a mutant in the BED domain were purified from BL21(DE3)pLysS *Escherichia coli* cells. Cells were grown in LD with 50 μg/ml Ampicillin at 30°C until OD_600nm_ = 0.5, then proteins expression was induced by adding 0.2 mM IPTG at 30°C for 3–4 h. For protein purification, cells were disrupted in Lysis buffer (300 mM NaCl, 50 mM sodium phosphate pH 7) with 30 mM imidazole by sonication (10 cycles 15 s ON/30 s ice). Protein extracts were clarified by centrifugation at 15 000 rpm for 1 h at 4°C. Clarified extracts were then loaded into a Ni-NTA Agarose (QIAGEN 1018244), matrix was washed with lysis buffer supplemented with 50 mM imidazole, then proteins were eluted in lysis buffer with 250 mM imidazole. Proteins were dialysed in 300 mM NaCl, 50 mM Tris–HCl pH 7.5, 10% Glycerol using Thermo Scientific™ Slide-A-Lyzer™ Dialysis Cassettes 3500 MWCO. The DNA sequences used in this study are reported in Supplementary Table S5. All oligonucleotides were purchased from Integrated DNA Technologies (IDT) with Ultramer synthesis. All buffers and reagents were purchased from Sigma-Aldrich and prepared according to common protocols using Milli-Q pure water. DNA probe strands were covalently immobilized on the surface of RPI sensing chips in spots having 150–200 μm diameter following the procedure described in (71). To allow the formation of G4 structures and coupling with the complementary sequence, the oligonucleotides were previously denatured by heating for 5 min at 95°C and renatured by slow cooling at room temperature in 10 mM Tris–HCl (pH 8.0), 100 mM KCl, 1 mM EDTA (pH 8.0). Before use, the BG4 antibody was suspended in measuring buffer A (20 mM Tris-HCl (pH 7.5), 1 mM DTT, 0.1 mg/ml BSA, 30 mM KCl) while Vid22 was suspended in measuring buffer B (20 mM Tris–HCl (pH 7.5), 1 mM DTT, 0.1 mg/ml BSA, 150 mM KCl). The RPI measurements were performed by using the apparatus and the analysis algorithm described in (71). The sensor cartridges were filled with 1.3 ml of measuring buffer A or B. The cartridges were kept at 25°C during the measurement by a thermalized holder and rapid mixing of the solution was provided by a magnetic stirring bar. Sample spikes of target BG4 or Vid22 were performed by adding 50 μl measuring buffer containing different amounts of target molecules to a final concentration in the cartridge of 0.08, 0.4, 2, 10 and 50 nM (only for Vid22). Time sequences of RPI images of the spotted surface were analyzed by a custom Matlab program in order to obtain the surface density of the target molecules *σ*(*t*) binding to the surface immobilized probes (71). The binding curves *σ_t_*(*t*) obtained by the RPI measurements were analyzed assuming a simple Langmuir model (72). The binding curves of at least six spots with identical composition were averaged and then fitted with an exponential growth function. Supplementary Table S6 reports the values for *K*_d_, *k*_on_ and *k*_off_ obtained from the fits of the curves in Figure 6B, E and Supplementary Figure S6D.

### UV spectrophotometric analysis

To collect information on the structure adopted by a DNA sequence, we measured and analyzed a series of thermal difference spectra of the oligos studied in this work. A thermal difference spectrum (TDS, (73)) can be computed subtracting the absorbance spectrum of a DNA oligonucleotide measured at low temperature (below melting) from the spectrum of the same oligonucleotide measured at high temperature (above melting). TDS recapitulates how temperature affects the spectral properties of the studied species and shows features that can be used to determine whether an oligonucleotide has transitioned from a G-quadruplex structure to a single stranded state upon thermal melting.

TDS is based on the widely studied hyperchromic effect of nucleic acids: the unstacking of nitrogenous bases results in an increase of their UV absorbance and this variation in the extinction coefficient is usually monitored to determine the melting temperature of oligonucleotides. This phenomenon effectively links the structure of the oligonucleotides to their absorbance and aside from monitoring the variation of amplitude at 260 nm, where the maximum typically lies, other wavelengths can be monitored to gather valuable information on the state in which the nitrogenous bases lie. While DNA duplexes show an increase of extinction coefficient at every typically measured wavelength when a melting experiment is performed, this is not true for G-quadruplex forming sequences: for the latter, as temperature increases above the G-quadruplex melting, the absorbance for wavelengths λ < 290 nm increases, while it decreases for λ > 290 nm (74). An isosbestic point in the TDS with value equal to zero at λ = 290 nm (black arrow in Figure 6C) corresponds to an inversion of temperature dependence of the absorbance and is a signature of G-quadruplex producing sequences.

All oligonucleotides used to measure TDS were purchased from Eurofins Genomics with no terminal modification using EXTREmers synthesis. The lyophilized powder was resuspended in Milli-Q pure water and for each DNA sequence a sample at ≈1 OD_260 nm_ was prepared with 100 mM KCl and degassed under vacuum. A series of complete spectra was acquired using a Thermo Scientific Evolution 300 UV-Vis Spectrophotometer in the 220–320 nm range using a 1 cm path quartz cuvette. The melting experiments were performed from 20°C to 90°C controlling the temperature with the instrument peltier module to obtain a rate of 0.4°C/min.

The signals of the thermal difference spectra (Δ*A*) were computed subtracting the spectrum measured at 20°C from the spectrum measured at 90°C and normalized for the maximum absorbance at low temperature (*A*_max_) to be directly compared and to highlight how a point mutation can heavily affect the behavior of Chr VIII-G4 sequence.

### DNA:RNA immunoprecipitation (DRIP) assays

DNA:RNA hybrids were immunoprecipitated using the S9.6 antibody from nucleic acid gently extracted and over- night digested with 50U of HindIII, EcoRI, BsrGI, XbaI and SspI, 2 mM spermidine and 2.5 ml BSA 10mg/ml genomic DNA; samples were then treated or not with RNase H, as described (75). Quantitative PCR was performed at the indicated loci. *SKN7* oligos (5′-CCG TTA ATT TCG CGA GCT TAT ACC TCA CCA TTC CAT TG-3′) and (5′-CCG TTA ATT AGC GCT GAT GTT GGA AGA TAG TAA GGT GA-3′). *GCN4* oligos (5′-TTG TGC CCG AAT CCA GTG A-3′) and (5′-TGG CGG CTT CAG TGT TTC TA-3′).

## RESULTS

### A genome-wide screen to identify new genes affecting genome stability

**Figure 1.**
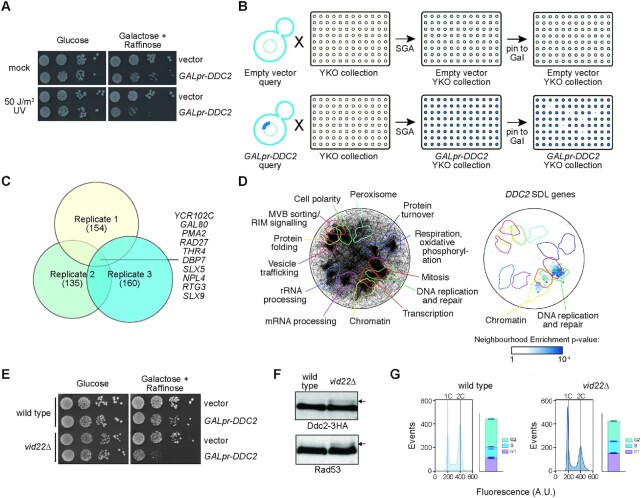
Genome-scale screen for synthetic dosage fitness defects with *DDC2* overexpression identifies *VID22*. Effect of *DDC2* overexpression on cell fitness. (**A**) Tenfold serial dilutions of exponentially growing wild-type cells carrying empty vector or *DDC2* under the control of the *GAL1* promoter were spotted on rich medium plates containing either glucose or galactose and raffinose as the carbon source. Cells were exposed to UV (50 J/m^2^), or mock-treated. Images were taken after three days of incubation at 28°C. (**B**) Schematic representation of the *DDC2* synthetic dosage lethality screen. The galactose-inducible *DDC2* gene (*GAL1pr-DDC2*), or the empty vector, was introduced into the yeast deletion collection (YKO) by crossing the collection with query strains containing the plasmids. Haploid strains containing each gene deletion and the plasmid were isolated using SGA methodology. Each strain was pinned to media containing galactose to induce *DDC2* expression. Synthetic dosage fitness defects were evident when colonies were smaller in *GAL1pr-DDC2* than in empty vector. (**C**) The overlap of the *DDC2* SDL genes for the three replicate screens is plotted as a Venn diagram. The number of positives in each replicate is indicated, as are the 10 genes identified in all three screens. (**D**) Spatial analysis of functional enrichment. On the left, the yeast genetic interaction similarity network is annotated with GO biological process terms to identify major functional domains (45). Thirteen of the 17 domains are labeled and delineated by colored outlines. On the right, the network is annotated with the 52 *DDC2* SDL genes. The overlay indicates the functional domains annotated on the left. Only nodes with statistically supported enrichments (SAFE neighborhood enrichment *P*-value < 0.05) are colored. (**E**) The *DDC2*–*VID22* SDL interaction is validated by growth analysis. Wild-type and *vid22Δ* cells were transformed with the empty vector or with the *GAL1pr-DDC2* plasmid. Tenfold serial dilutions of exponentially growing cultures were spotted on glucose and galactose plus raffinose plates to repress or induce Ddc2 overexpression, respectively. Images were taken after three days of incubation at 28°C. (**F**) Spontaneous DNA damage checkpoint activation was evaluated by monitoring phosphorylation of the checkpoint proteins Ddc2 (Ddc2-3HA) and Rad53. Protein extracts from wild-type or *vid22Δ* cells expressing Ddc2-3HA were analyzed by western blotting with anti-HA and with anti-Rad53 antibodies. Arrows indicate the phosphorylated forms of Ddc2 and Rad53. (**G**) The cell cycle distribution of exponentially growing cultures was determined by flow cytometry of logarithmic phase wild-type (G1-25.9%; S-19%; G2-54.2%) and *vid22*Δ (G1-34.3%; S-22.4%; G2-38.3%) cultures from three independent replicates. The positions of cells with 1C and 2C DNA contents are indicated.

*S. cerevisiae* Ddc2 is a binding partner and activator of the DNA damage checkpoint (DDC) apical kinase Mec1 (human ATR) (76). *DDC2* overexpression has only a minor effect in unperturbed cells, while it causes hyperactivation of the checkpoint response after DNA damage, leading to prolonged cell cycle arrest and increased cell lethality ((42) and Figure 1A). We hypothesized that yeast mutants spontaneously accumulating DNA damage might be sensitized to *DDC2* overexpression (77), and therefore would show synthetic dosage fitness defects (78) with *DDC2*. We developed a screen to identify genes and pathways involved in genome integrity maintenance based on their sensitivity to *DDC2* overexpression. Using Synthetic Genetic Array (SGA) technology (43,79), we introduced a multicopy plasmid carrying *DDC2* under the control of a galactose-inducible promoter, and a control empty vector, into the yeast knockout (YKO) collection (Figure 1B). We identified mutants that, on galactose-containing medium, exhibited reduced fitness, often referred to as synthetic dosage lethality (SDL), when the *DDC2*-containing plasmid was present compared to controls with the empty vector. From three independent replicate screens, we identified 52 genes that were positive in at least two replicates (Figure 1C and Table 1). Consistent with our initial hypothesis, several of these 52 genes were previously identified in at least one systematic screen for increased spontaneous DNA damage (35,80–82).

**Table 1.** *DDC2* synthetic dosage lethality genes

Genes with epsilon < –0.08 in at least 2 replicates^a^	Systematic name	Replicate 1 epsilon^b^	Replicate 2 epsilon	Replicate 3 epsilon	Mean epsilon	Std Dev	Brief description^c^
***YCR102C***	*YCR102C*	−0.34593	−0.26015	−0.22037	−0.2755	0.0524	Putative quinone oxidoreductase
***GAL80***	*YML051W*	−0.30714	−0.16683	−0.25393	−0.2426	0.0578	Transcriptional regulator involved in the repression of GAL genes
*PYC2*	*YBR218C*	0	−0.38475	−0.23274	−0.2058	0.1582	Pyruvate carboxylase isoform
***PMA2***	*YPL036W*	−0.25367	−0.11122	−0.1438	−0.1696	0.0609	Plasma membrane H+-ATPase
***RAD27***	*YKL113C*	−0.16946	−0.08576	−0.16575	−0.1403	0.0386	5′ to 3′ exonuclease, 5′ flap endonuclease
*VID22*	*YLR373C*	−0.07129	−0.10449	−0.21956	−0.1318	0.0635	Glycosylated integral membrane protein localized to plasma membrane
*MMS22*	*YLR320W*	−0.13772	−0.00623	−0.23168	−0.1252	0.0925	Subunit of E3 ubiquitin ligase complex involved in replication repair
*VPS68*	*YOL129W*	−0.02521	−0.23802	−0.10864	−0.1240	0.0876	Vacuolar membrane protein of unknown function
***THR4***	*YCR053W*	−0.12709	−0.11031	−0.12391	−0.1204	0.0073	Threonine synthase
***DBP7***	*YKR024C*	−0.08875	−0.16635	−0.10059	−0.1186	0.0341	Putative ATP-dependent RNA helicase of the DEAD-box family
***SLX5***	*YDL013W*	−0.112	−0.1192	−0.12084	−0.1173	0.0038	Subunit of the Slx5-Slx8 SUMO-targeted Ub ligase (STUbL) complex
*LRO1*	*YNR008W*	0.00822	−0.15022	−0.19615	−0.1127	0.0875	Acyltransferase that converts diacylglycerol to triacylglycerol (TGA)
*NKP2*	*YLR315W*	−0.21546	−0.02581	−0.09278	−0.1114	0.0785	Central kinetochore protein and subunit of the Ctf19 complex
***NPL4***	*YBR170C*	−0.10522	−0.12311	−0.10379	−0.1107	0.0088	Substrate-recruiting cofactor of the Cdc48p-Npl4p-Ufd1p segregase
*SST2*	*YLR452C*	−0.15523	−0.1565	−0.01984	−0.1105	0.0641	GTPase-activating protein for Gpa1p
*IRC4*	*YDR540C*	−0.1842	−0.02255	−0.106	−0.1043	0.0660	Protein of unknown function
*RPL22A*	*YLR061W*	−0.12007	0.00518	−0.19649	−0.1038	0.0831	Ribosomal 60S subunit protein L22A
*SCS22*	*YBL091C-A*	−0.09108	−0.07698	−0.13178	−0.0999	0.0232	Protein involved in regulation of phospholipid metabolism
*EKI1*	*YDR147W*	−0.12455	−0.07955	−0.09191	−0.0987	0.0190	Ethanolamine kinase
*SLX8*	*YER116C*	−0.15284	0.01681	−0.15558	−0.0972	0.0806	Subunit of Slx5-Slx8 SUMO-targeted ubiquitin ligase (STUbL) complex
*MNN10*	*YDR245W*	−0.09849	−0.11437	−0.07617	−0.0963	0.0157	Subunit of a Golgi mannosyltransferase complex
*PTC4*	*YBR125C*	−0.23321	−0.11234	0.05822	−0.0958	0.1196	Cytoplasmic type 2C protein phosphatase (PP2C)
***RTG3***	*YBL103C*	−0.09982	−0.08377	−0.09895	−0.0942	0.0074	bHLH/Zip transcription factor for retrograde (RTG) and TOR pathways
*PAC10*	*YGR078C*	0.0254	−0.13636	−0.16526	−0.0921	0.0839	Part of the heteromeric co-chaperone GimC/prefoldin complex
***SLX9***	*YGR081C*	−0.0811	−0.08407	−0.10933	−0.0915	0.0127	Protein required for pre-rRNA processing
*GUP1*	*YGL084C*	−0.02287	−0.15367	−0.09719	−0.0912	0.0536	Plasma membrane protein involved in remodeling GPI anchors
*KCC4*	*YCL024W*	−0.13964	−0.08448	−0.04869	−0.0909	0.0374	Protein kinase of the bud neck involved in the septin checkpoint
*MRS2*	*YOR334W*	−0.1369	−0.15801	0.02559	−0.0898	0.0820	Mitochondrial inner membrane Mg(2+) channel
*RTS1*	*YOR014W*	−0.06842	−0.09715	−0.09979	−0.0885	0.0142	B-type regulatory subunit of protein phosphatase 2A (PP2A)
*TIM18*	*YOR297C*	0.09083	−0.14955	−0.20461	−0.0878	0.1283	Component of the mitochondrial TIM22 complex
*SAC7*	*YDR389W*	−0.09922	−0.07025	−0.08967	−0.0864	0.0121	GTPase activating protein (GAP) for Rho1p
*HTZ1*	*YOL012C*	−0.12843	−0.09077	−0.03809	−0.0858	0.0371	Histone variant H2AZ
*CTF4*	*YPR135W*	−0.00526	−0.10066	−0.14951	−0.0851	0.0599	Chromatin-associated protein
*LYS2*	*YBR115C*	0	−0.13134	−0.1208	−0.0840	0.0596	Alpha aminoadipate reductase
*YDL162C*	*YDL162C*	−0.01668	−0.08016	−0.1529	−0.0832	0.0557	Overlaps with CDC9 promoter
*MRC1*	*YCL060C*	−0.10752	−0.05301	−0.08651	−0.0823	0.0224	S-phase checkpoint protein required for DNA replication
*PHO5*	*YBR093C*	−0.14925	−0.12743	0.03574	−0.0803	0.0825	Repressible acid phosphatase
*MRX10*	*YDR282C*	−0.09352	−0.0926	−0.04064	−0.0756	0.0247	Mitochondrial inner membrane protein of unknown function
*RPL40A*	*YIL148W*	0.10568	−0.16715	−0.16321	−0.0749	0.1277	Ubiquitin-ribosomal 60S subunit protein L40A fusion protein
*XDJ1*	*YLR090W*	0.0167	−0.08185	−0.15132	−0.0722	0.0689	Chaperone with a role in facilitating mitochondrial protein import
*YNL140C*	*YNL140C*	−0.02494	−0.08911	−0.10192	−0.0720	0.0337	Protein of unknown function
*PEX9*	*YMR018W*	−0.09397	−0.09198	−0.02484	−0.0703	0.0321	Peroxisomal membrane signal receptor for peroxisomal matrix proteins
*UBP6*	*YFR010W*	−0.09206	−0.00378	−0.11453	−0.0701	0.0478	Ubiquitin-specific protease
*DST1*	*YGL043W*	−0.09769	0.00767	−0.11659	−0.0689	0.0547	General transcription elongation factor TFIIS
*SPO73*	*YER046W*	−0.12322	−0.09846	0.02037	−0.0671	0.0627	Meiosis-specific protein required for prospore membrane morphogenesis
*BRE5*	*YNR051C*	−0.13273	−0.1059	0.0386	−0.0667	0.0752	Ubiquitin protease cofactor
*MCA1*	*YOR197W*	−0.0821	−0.10252	−0.00329	−0.0626	0.0428	Ca^2+^-dependent cysteine protease
*SIF2*	*YBR103W*	0.01601	−0.09958	−0.09643	−0.0600	0.0538	WD40 repeat-containing subunit of Set3C histone deacetylase complex
*TOS4*	*YLR183C*	0.05837	−0.12147	−0.10298	−0.0554	0.0808	Putative transcription factor, contains Forkhead Associated domain
*YML090W*	*YML090W*	0.04237	−0.09205	−0.11481	−0.0548	0.0694	Dubious open reading frame
*SCS7*	*YMR272C*	0.20929	−0.15347	−0.0807	−0.0083	0.1567	Sphingolipid alpha-hydroxylase
*ASK10*	*YGR097W*	0.59527	−0.11235	−0.10438	0.1262	0.3317	Regulator of the Fps1p glycerol channel

^a^Genes identified in all three replicates are indicated in **bold**.

^b^Epsilon values with *P* > 0.05 are entered as ‘0’.

^c^As annotated in YeastMine (https://yeastmine.yeastgenome.org/).

Twelve genes were previously reported to exhibit synthetic dosage growth defects when combined with *DDC2* overexpression (*Saccharomyces* Genome Database, https://www.yeastgenome.org, accessed 12 July 2020). These include *HOG1*, *RPD3* and *SLT2* (83,84) and *DUN1* (85), which were identified in screens where the given deletion mutant was the query. An SDL screen in a pooled format with *GA1Lpr-DDC2* as the query identified *ASF1*, *CSM3*, *CTF4*, *MMS1*, *RAD27*, *RTT101*, *RTT109* and *TOF1* (86). Of these 12 genes, *RAD27* and *CTF4* were identified in our screens. Thus, we identified 50 putative synthetic dosage interactions that had not been reported previously.

To assess the functional properties of the 52 gene *DDC2* SDL set, we applied spatial analysis of functional enrichment (SAFE) (44) to determine if any regions of the functional genetic interaction similarity yeast cell map (45) are over-represented for the SDL gene set (Figure 1D). We found a statistically supported over-representation of the *DDC2* SDL genes in the DNA replication and repair neighborhood of the genetic interaction cell map, indicating that defects in DNA replication and repair sensitize cells to *DDC2* overexpression. Over-representation of the *DDC2* SDL genes in the chromatin and transcription neighborhoods of the genetic interaction cell map was also evident, consistent with a role for *DDC2* in sensing chromatin dysfunction.

### *VID22* suppresses spontaneous DNA damage

Of the 52 genes identified in our screens, we focused our attention on *VID22*. Although this gene shows conflicting functional annotations, including vacuole import and degradation (87), it was previously identified also in other genetic screens searching for players involved in genome integrity maintenance (87–89). *VID22* encodes a nuclear protein (31,90) computationally predicted to contain a BED-type zinc finger domain and a RNaseH-like domain (32,33) and it was suggested to play a role in DNA DSB repair (34,35).

Previous observations in strains experiencing spontaneous DNA damage (29,91,92)—as well as the rationale behind our genetic screen—led us to suspect that, in a *vid22* mutant, the DDC might be chronically activated even in the absence of external genotoxic insults albeit to a low level. After confirming the synthetic dosage lethality due to *DDC2* overexpression in a *vid22Δ* strain (Figure 1E), we verified our hypothesis that both Ddc2 and Rad53, markers of DDC activation (93,94), are partially phosphorylated in unperturbed *vid22Δ* cells (Figure 1F), similarly to other mutants known to spontaneously accumulate genomic instability such as *slx5Δ* and *slx8Δ* that also emerged in our SDL screen (Supplementary Figure S1A, (77) and Table 1). Thus, loss of *VID22* causes spontaneous activation of the DNA damage checkpoint response, which is compatible with an increased formation of spontaneous DNA lesions. Consistently with a previous screen (95), analysis of the cell cycle distribution of *vid22Δ* mutants shows a significant accumulation in the G1 subpopulation (Figure 1G). This observation excludes the possibility that Ddc2 and Rad53 phosphorylation is due to a G2/M accumulation (76), supporting instead the notion that the checkpoint response might be chronically alerted as a consequence of the elevated spontaneous formation of endogenous DNA damage. We further investigated whether the accumulation of cells in G1 observed in Figure 1G may be due to this chronic activation of the DDC, delaying entry into S-phase. However, ablation of the checkpoint kinase Mec1 does not rescue this defect, suggesting that the cell cycle delay observed in *vid22Δ* mutants may be, at least partially, of different origin (Supplementary Figure S1B).

### Loss of *VID22* leads to chromosomal alterations

**Figure 2.**
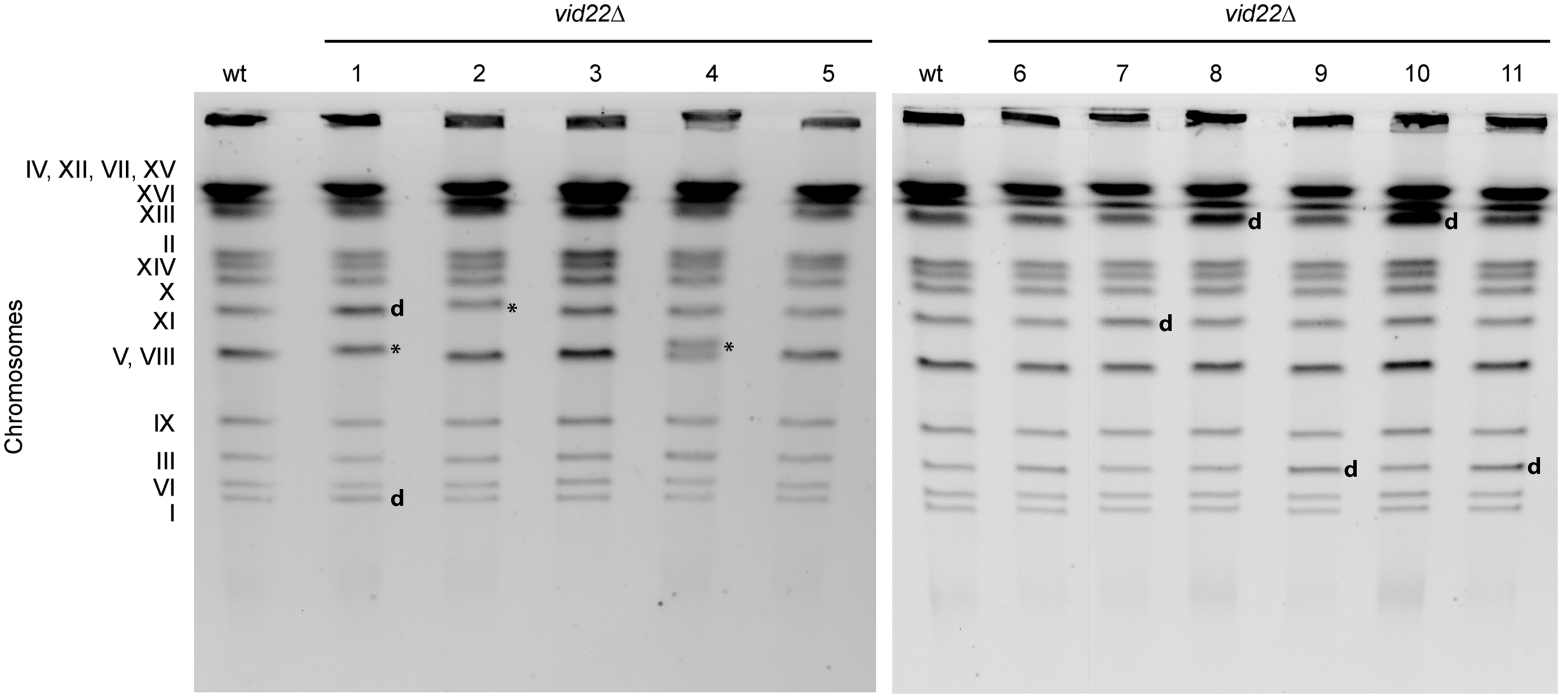
*vid22*Δ cells accumulate gross chromosomal rearrangements and chromosome duplications. DNA was prepared from wild-type and eleven independent *vid22Δ* mutants and chromosomes were fractionated by pulsed-field gel electrophoresis. The positions of the 16 chromosomes are indicated on the left. * indicates chromosome size changes; **d** indicates chromosome duplications.

Chronic exposure to endogenous DNA damage frequently leads to genomic instability. To assess whether loss of *VID22* causes the accumulation of chromosomal aberrations, we analyzed the chromosomes of 11 independent unperturbed *vid22Δ* clones by PFGE and compared them to wild-type strains (Figure 2 and Supplementary Figure S2A). Eight of the eleven *vid22Δ* clones exhibited at least one detectable chromosomal aberration (Figure 2), which did not occur in the 10 wild-type control clones (Supplementary Figure S2A). In *vid22Δ* mutants we identified either entire chromosomal duplications (e.g. Chr XI and I in strain 1) or increased chromosome length (e.g. Chr XI in strain 2 or Chr V or VIII in strain 4) (Figure 2). To exclude that the observed abnormalities might be due to different numbers of cell generations, we repeated the analysis starting from the sporulation of a heterozygous diploid *VID22*/*vid22Δ*. As shown in Supplementary Figure S2B, after the same number of cell divisions, 4 of 7 *vid22Δ* mutants show at least 1 major chromosomal abnormality, not observed in any of the 13 wild-type clones tested.

**Figure 3.**
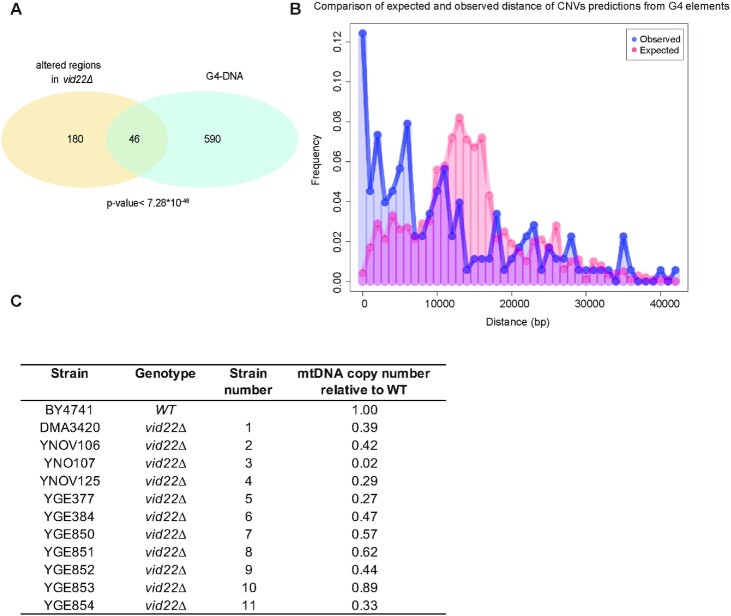
Genome sequence analysis of *vid22Δ* mutants reveals preferential genome instability at G4-rich loci. (**A**) The intersection of predicted G4 elements (17) with small (less than 2 Kb) copy number alterations detected by genome sequencing of eleven *vid22Δ* mutants. The overlap of chromosome coordinates of small CNVs detected in genome shotgun sequences of *vid22Δ* mutants and G4 elements in the yeast genome predicted by (17) are plotted as a Venn diagram. The indicated *P*-value of the intersection is from the Fisher's exact test. (**B**) Comparison of the distribution of expected an observed distance of CNV predictions from G4 elements. Frequency distribution of observed distance of predicted CNV from G4 elements is represented in blue. The expected distance distribution, estimated by 1000 independent random resampling of a matched number of genomic windows of identical size, is represented in red. Distances in base pairs (bp) are represented on the X axis, frequencies on the Y axis. (**C**) The inferred mtDNA copy number of eleven *vid22Δ* mutant strains. The copy number of mtDNA relative to the wild-type strain was inferred by comparing ratios of mitochondrial read counts, using quantile normalization and GC content normalization.

To investigate whether particular chromosomal or sequence contexts are associated with the observed genome instability, we sequenced the genomes of the 11 *vid22Δ* strains shown in Figure 2. While *de novo* assemblies of the mutant strains’ genomes did not indicate the presence of large structural genomic alterations (insertions, inversion, or translocations), prediction of Copy Number Variants (CNVs) based on the depth of coverage analyses were consistent with complete duplications for chromosomes I (strain 1), III (9 and 11), XI (1 and 7) and XIII (8 and 9). In addition, we observed 468 smaller CNVs not detected by PFGE, associated with 226 distinct genomic loci (Supplementary Table S7). Importantly, patterns and the total number of observed single nucleotide substitutions and small indels (less than 5 base pair in size) to the reference assembly of the *S. cerevisiae* genome, were highly consistent between wild type BY4741 and *vid22* mutant strains (Supplementary Table S8), with only a marginal increase observed in strain 9. Overall this observation might suggest that *vid22* mutants are not systematically associated with an increased mutational burden and/or systematic defects in DNA repair mechanisms. Intriguingly, the CNVs detected from genome sequencing of *vid22*Δ strains show both statistically supported overlap and an association with G4 elements predicted by Capra and colleagues (17) (Figure 3A and B, hypergeometric *P* < 7.28E–48), suggesting that disruption of *VID22* is linked to increased genomic instability at G4-DNA rich genomic regions. In addition, overlap between the observed CNVs and G4s is still significant when interrogating different databases of predicted G4 regions (Supplementary Tables S9, S10 and Figure S3 (17,96,97)). Consistent with this model, we observed that *VID22* deletion induces great instability of the mitochondrial genome, which is particularly rich in potential G-quadruplex forming regions (17) (Figure 3C).

### *VID22* maintains telomere length

**Figure 4.**
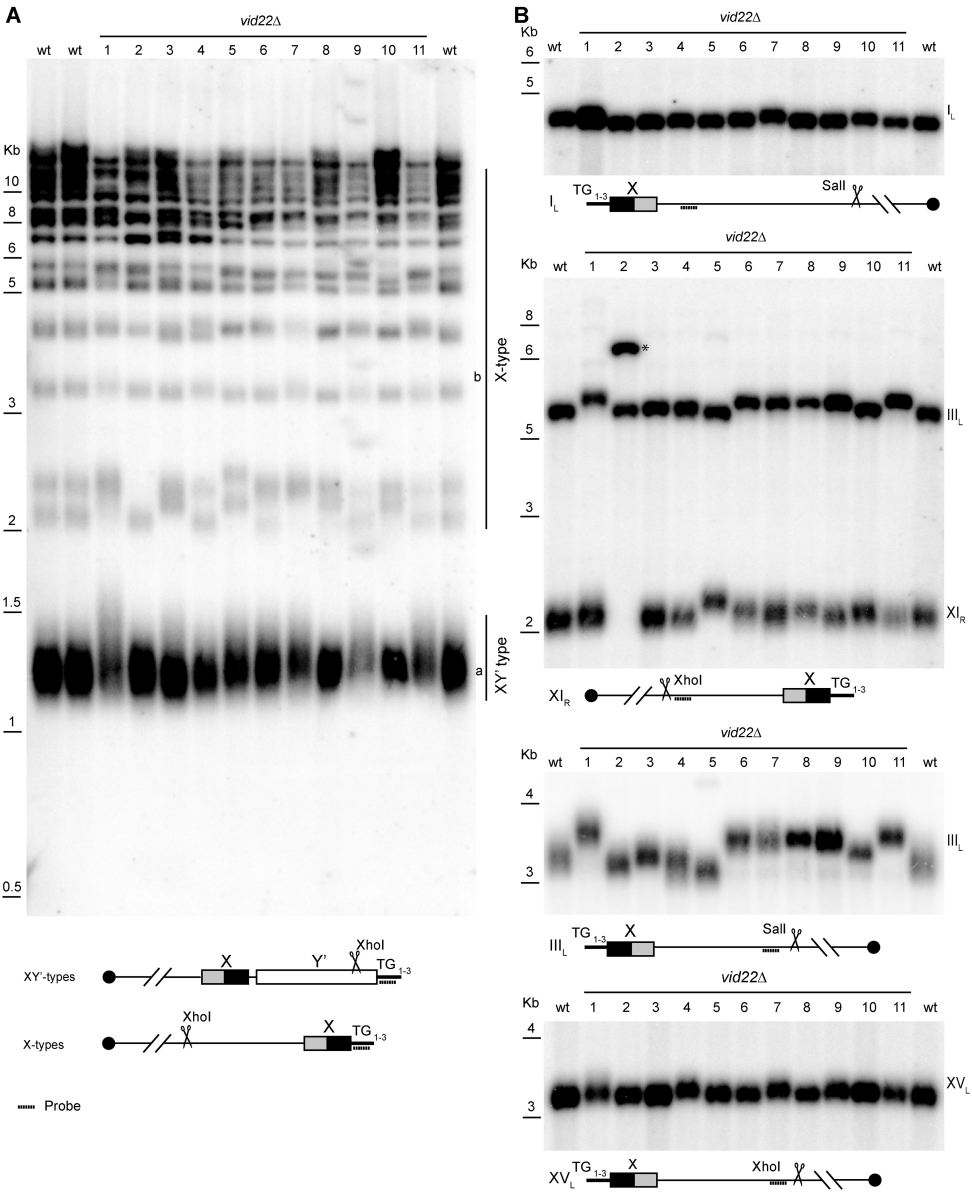
Vid22 is important for the maintenance of telomere homeostasis. Genomic DNA was prepared from wild-type and the eleven independent *vid22Δ* strains. DNA was digested with XhoI or SalI, fractionated on agarose gel, and hybridized with the indicated probes. (**A**) Southern blot and schematic for XY’-type telomeres. (**B**) Southern blot and schematic for 4 different X-type telomeres: I_L_, XI_R_, III_L_, XV_L_. The probe for the XI_R_ telomere cross-reacts with telomere III_L_ (as described in (56)). The band corresponding to XI_R_ of clone number 2 is indicated with *.

Telomeres are among the regions that show the highest density of sequences potentially forming G4-DNA structures (17,98). Budding yeast telomeres terminate with repetitive TG_1–3_ ends and contain an adjacent X core conserved region; an additional Y’ subtelomeric region is also present at several telomeres, as schematized in Figure 4 (99,100). Telomeric sequences are highly repetitive, hampering their analysis by short sequencing reads. We directly monitored the telomeres of the 11 *vid22Δ* mutants using a Southern blot approach. Digestion of genomic DNA with XhoI generates a population of TG_1–3_ repeats of XY’ type telomeres with a size of 1–1.5 kb (Figure 4A). In the X-only telomeres, XhoI cuts in variable positions, generating a wide spectrum of digestion fragments with sizes between 3 and 12 kb ((56) and Figure 4A). Using a TG_1–3_ specific probe, we found that the deletion of *VID22* generally causes an increase in the average length of telomeric ends (Figure 4A). This phenomenon is clearly detected in the XY’-type telomeres (bottom of panel 4A), and is also evident in the X-only fragments (top of the panel 4A). To detect X-only telomeres specifically, we used the strategy described in (56) and schematized in Figure 4B, which allowed us to visualize I_L_, III_L_, XI_R_ and XV_L_ telomeres. As shown in Figure 4B, in the absence of *VID22*, we were able to distinguish telomere alterations that are particularly evident at telomeres III_L_ and XI_R_.

### *VID22* suppresses G4-dependent genome instability

**Figure 5.**
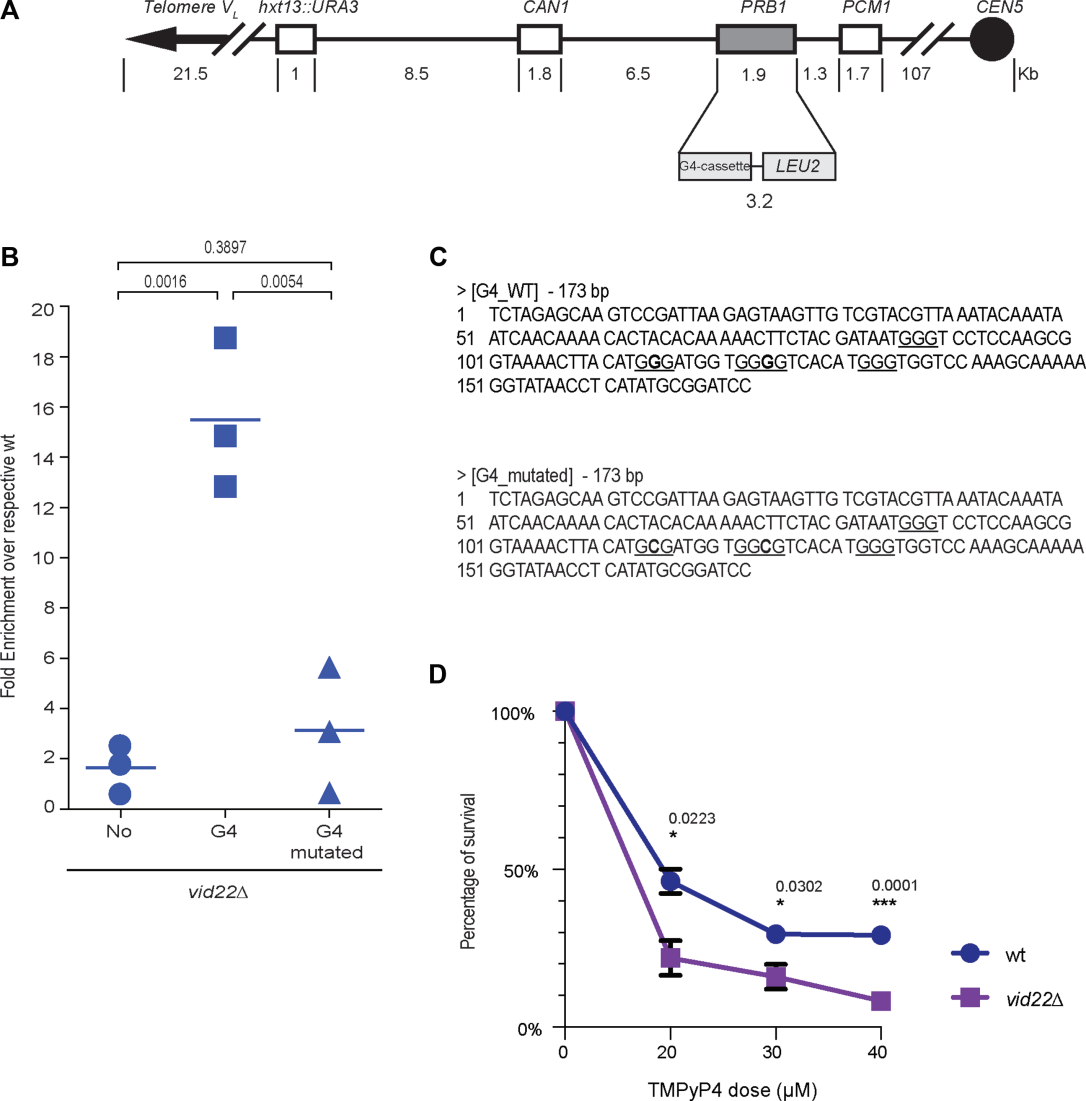
Gross chromosomal rearrangements and cell lethality induced by G4 DNA in *vid22*Δ. (**A**) Schematic representation of the left arm of Chr V in strains used for GCR assay. The G4 or the G4-mutated cassettes were inserted at the *PRB1* locus. *PCM1* is the essential gene nearest to the left telomere; *URA3* and *CAN1* are the two genetic markers used to select for chromosome arm loss or interstitial deletions. (**B**) The plot represents the fold enrichment obtained with GCR experiments. The fold enrichment is the ratio of the GCR rate between *vid22Δ* and wild type containing the same cassette. Each data point is from an independent fluctuation test, with *n* ≥ 3 for each strain. The horizontal bars indicate the mean CGR rate for each strain (*N* = 3 independent experiments). An unpaired Student's *t*-test was used to compare the means of measurements and the p-value is indicated. (**C**) Partial sequences of the two cassettes inserted at *PRB1* locus. The underlined Gs are essential for G4-DNA formation; bold Gs in G4-mutated sequence were substituted with C to abolish G4 formation. (**D**) Survival curve after TMPyP4 treatment. Wild-type and *vid22Δ* strains were treated for 2 hours with TMPyP4 at the indicated concentration and plated on YEPD. The percentage of survival was reported (*N* = 3 independent experiments). An unpaired Student's *t*-test was used to compare the means of measurements and the p-value is indicated.

To explore the hypothesis that *vid22Δ* mutants are susceptible to genome instability in the presence of G4-DNA structures, we exploited an assay for gross chromosomal rearrangements (GCRs) (21,101). Briefly, we used a modified chromosome V that harbours the *CAN1* and *URA3* markers in the left arm (Figure 5A). This configuration allows simultaneous selection on canavanine and 5-FOA to identify chromosome arm loss and large interstitial deletions. To analyse G4-induced GCRs, we inserted a cassette predicted to form G4-DNA (21) at the *PRB1* locus on the modified chromosome and monitored the GCR frequency. When a G4-forming motif is employed, deletion of *VID22* leads to a 15-fold increase in GCRs relative to the corresponding wild-type strain (Figure 5B). By contrast, deletion of *VID22* has little effect when the *PRB1* locus is unchanged (Figure 5B). To test whether this effect was indeed caused by the G4 structure, we mutagenized the G4 motif by substituting two GGGs with GCGs (Figure 5C), maintaining the same GC content but reducing the sequence propensity to form G4 structures. When the G4 mutated motif was introduced at the *PRB1* locus, we did not observe a statistically supported difference in GCRs between the *vid22Δ* and wild-type strain (Figure 5B), indicating that the chromosomal instability observed in the absence of *VID22* strain is strongly related to the presence of G4 structures.

We then tested the sensitivity of the *vid22Δ* mutant to TMPyP4, a widely used G4-binding molecule that causes stabilization of these nucleic acid structures (36,102). As shown in Figure 5D, deletion of *VID22* significantly enhances cell sensitivity to the G4-ligand, strongly corroborating the hypothesized role of Vid22 in controlling G4 stability.

In yeast cells, a Vid22 paralog, Env11, is present (29). Since frequently paralogs exhibit partially overlapping functions, we tested whether Env11, which also interacts with Vid22, is similarly implicated in preserving genome stability at G4-prone regions. In the *env11Δ* mutant, the G4 dependent GCR rate is indistinguishable from that of a wild type (Supplementary Figure S4A). Moreover, *ENV11* deletion does not affect the sensitivity of the *vid22Δ* mutant to replication stress agents as Hydroxyurea or MMS (Supplementary Figure S4B and (34)), suggesting that Vid22 plays a specific role in maintaining genome stability, independently of *ENV11*.

### Vid22 binds to and controls the stability of G4 regions

To determine the *in vivo* sites that were bound by Vid22, we performed a Vid22 ChIP-seq analysis. A total of 413 ChIP-seq peaks were recovered, and in agreement with data published in Styles *et al.* (35) they are preferentially associated with gene promoters (Fisher Exact test *P*-value 1.49E–07) and significantly enriched (∼ 2 fold) in the proximity (0 and 200 bp) of predicted G4 elements (Fisher exact test *P*-value 1.03E–06) (Supplementary Table S11 and Supplementary Figure S5). Importantly, the levels of enrichment in G4 elements were consistent across different types of annotations (TSS, promoters, exons) (Supplementary Figure S5A), suggesting that the preferential co-localization of Vid22 with G4 is not associated with specific genomic features.

**Figure 6.**
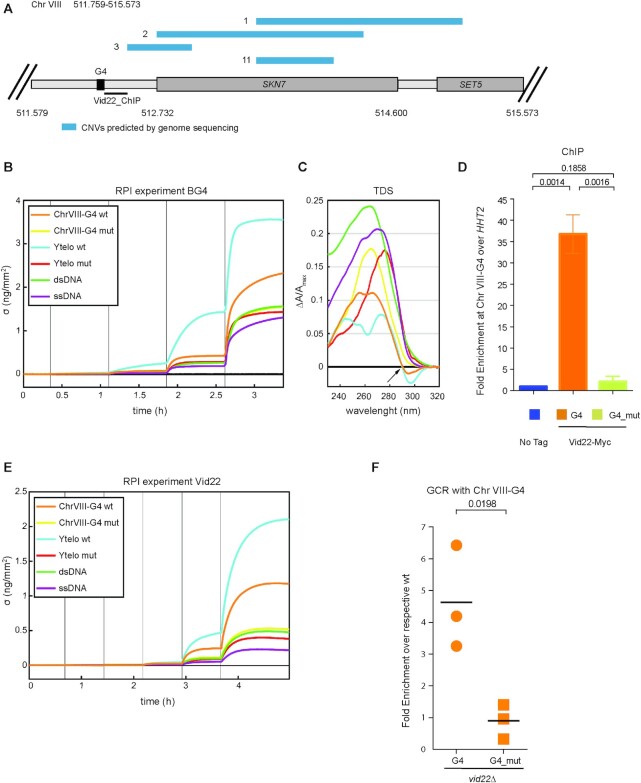
Vid22 binds to and controls the stability of Chr VIII-G4 region. (**A**) Graphical representation of genomic features associated with the Chr VIII *511.759–515.573* genomic locus. A G4 element predicted by (17) is indicated. Copy Number Variations (CNVs) detected at this region in *vid22Δ* strains in this study are indicated with blue bars. The region analyzed by qPCR for ChIP is indicated with a solid black line. (**B**) *In vitro* ability of the predicted Chr VIII-G4 sequence to form G4 structure. Analysis of the interaction of the BG4 antibody with different DNA sequences using the Reflective Phantom Interface (RPI) technique: control G4-forming sequence (Ytelo) and the mutated form Ytelo mut (123), unrelated ssDNA or dsDNA sequences, ChrVIII-G4 and ChrVIII-G4 mut. Increase of the surface density (*σ*) was measured over time upon antibody binding for the rising concentration of BG4 in solution (*T* = 25°C, and *c* = 10 μM). The vertical lines mark the time where BG4 concentration was increased step-wise from 0 to 10 nM. (**C)** Thermal difference spectra (TDS) of sequences used in the RPI experiment. Each spectrum is computed subtracting the absorbance spectrum at 20°C from the absorbance spectrum at 90°C and then normalized for the maximum amplitude at low temperature to allow a direct comparison among the different sequences. A neat isosbestic point associated with G-quadruplex forming species at 290nm is present (black arrow) both in Ytelo and ChrVIII-G4, while being absent in every other species studied. Y axis is stretched to fit the high TDS amplitude of ChrVIII-G4 mut, ssDNA and dsDNA. (**D**) ChIP-qPCR of Vid22 at the G4 predicted region (17) at the Chr VIII *SKN7* locus. ChIP was performed in wild type (No Tag), Vid22-13Myc (G4) and Vid22-13Myc harbouring two point mutations in the G4 predicted region (G4-mutated). Fold enrichment of Vid22 at Chr VIII was calculated relative to the internal standard *HHT2*. Data are represented as mean ± SEM of *N* = 3 independent experiments. (**E**) Analysis of interaction of Vid22 with DNA forming G4 structures using the Reflective Phantom Interface (RPI) as reported in panel B. Vid22 concentration was increased step-wise from 0 to 50 nM. (**F**) The plot represents the fold enrichment obtained with GCR assays in which *PRB1* locus was substituted with Chr VIII region (Figure 5A). The fold enrichment is the ratio of the GCR rate between *vid22Δ* and wild-type strain containing the same cassette. Each data point is from an independent fluctuation test, with *n* ≥ 3 for each strain. The horizontal bars indicate the mean GCR rate for each strain (*N* = 3 independent experiments). (D–F) An unpaired Student's *t*-test was used to compare the means of measurements and the p-value is indicated.

Analyzing genomic sequencing data described above, we could identify the rearrangement hotspots in *vid22Δ* cells. We found that the *SKN7* locus, located on Chr VIII, is altered in 4 of 11 strains sequenced (Supplementary Table S7 and scheme Figure 6A). Consistently with our data, the region located upstream of *SKN7* is predicted to form G4 structures ((17) and Supplementary Figure S6A) and was enriched in our ChIP-seq analysis (Supplementary Table S11) and in Styles *et al.* (35).

To confirm experimentally the ability of the *SKN7* upstream sequence to form G4 structures *in vitro*, we analyzed the conformation of synthetic DNA sequences by UV spectrophotometric analysis and monitored binding of the G4-specific BG4 antibody by Reflective Phantom Interface (RPI). We compared a wild-type *SKN7* sequence (ChrVIII-G4 wt) to a mutant *SKN7* (ChrVIII-G4 mut) that was altered in its predicted capacity to form a G4 structure. As controls, we included a yeast telomeric sequence (Ytelo wt), known to form G4s, a mutated Ytelo, a ssDNA sequence and a dsDNA sequence. The results shown in Figure 6B-C and Supplementary Table S5, demonstrate that Ytelo wt and ChrVIII-G4 wt do indeed form G4 structures, as revealed by the absorbance spectrum and by the binding of the BG4 antibody, as detected by RPI analysis. Both features were lost in the mutated sequences.

We next directly tested *in vivo* binding of Vid22 at the *SKN7* genomic site by ChIP analysis and found that Chr VIII-G4 nt 512387–512565 is enriched in Vid22 chromatin immunoprecipitates (Figure 6D). Vid22 binding to Chr VIII-G4 *in vivo* is dependent on the ability of the target sequence to form G4 structures. Indeed, when we converted the chromosomal G4-forming sequence to a mutated version that loses the ability to form G4s (Supplementary Figure S6A and Figure 6B-C), binding of Vid22, as measured by ChIP, was also lost (Figure 6D).

It is important to note that two consensus sites for Tbf1 binding (TAGGG) (30) are present in proximity of the *SKN7* G4-forming sequence (Supplementary Figure S6A). Thus, we cannot exclude that the observed Vid22 chromatin enrichment might depend on the binding of Tbf1 to these sites. However, mutation of both Tbf1 consensus sites in TA nucleotides ((103) and Supplementary Figure S6A) does not significantly alter the ability of Vid22 to bind this region, which is still totally dependent on the G4 structure (Supplementary Figure S6B).

By ChIP, we excluded that the observed Vid22 binding is mediated by the Sgs1 protein (Supplementary Figure S6C), as was previously observed for other loci (35).

To verify that Vid22 can directly bind G4 structures *in vitro*, we analyzed by RPI the binding of recombinant Vid22 purified from *E. coli* to the synthetic sequences described above. As shown in Figure 6E, Vid22 shows a strong affinity to Ytelo and *SKN7* G4-forming sequences; the binding depends on the sequences’ ability to fold into G4, as it is lost when Ytelo or SKN7 are mutated. Surprisingly, Vid22 direct binding to G4s is not mediated by the protein's BED domain as the interaction is not altered by mutation of the conserved CCHH signature (32) (Supplementary Figure S6D).

To verify that Vid22 binding to this G4-forming sequence is important to protect Chr VIII from GCRs, we introduced the wild-type or mutated *SKN7* G4 region into the *PRB1* locus in the GCR reporter strain and measured GCR rates in wild-type and *vid22Δ* strains. Deletion of *VID22* induces nearly five times more rearrangements in proximity to the *SKN7* G4 region compared to a *VID22* wild-type strain and this instability is dependent upon G4 formation, similarly to what was observed for Vid22 binding (Figure 6F). Overall, these data indicate that Vid22 binds regions that are likely to form physiological G4-DNA structures and counteracts G4-induced genome instability.

## DISCUSSION

Genomic instability is a hallmark of cancer (9,11,104) and it has been proposed to act as a key driving force in tumorigenesis (12,104,105). Given the evolutionary conservation of the pathways that preserve DNA integrity, yeast research has provided valuable insights into the regulation of genome stability in humans. Multiple screens in *S. cerevisiae* have identified crucial players in genome stability maintenance (80,82,106–114). Nonetheless, the results of these screens are only partially overlapping, likely due to the different approaches used, and each study provides a distinct perspective of the genome integrity network. To identify new genes implicated in protecting cells from endogenous DNA damage, we used a modified version of the SGA technology (79), and screened the *S. cerevisiae* deletion collection for mutants that exhibited chronic accumulation of spontaneous DNA damage. The strategy was based on the observation that overexpression of the cell cycle protein Ddc2 in cells experiencing DNA damage causes hyperactivation of the DNA damage checkpoint and impaired growth ((42) and Figure 1A). We exploited the sensitivity to Ddc2 overexpression as a readout for the spontaneous accumulation of DNA lesions that activate the DNA damage checkpoint, thus broadening the range of genomic instability marks detected in a single screen.

In our screens, we identified two genes with reported synthetic dosage growth defects when combined with *DDC2* overexpression and 50 additional genes not previously known to have SDL interactions with *DDC2*. The 52 genes identified in our screens included 18 genes known to associate with genome instability phenotypes, and were enriched for genetic interactions in the DNA replication and DNA repair neighbourhood of the genetic interaction cell map (45), indicating that genes displaying an SDL interaction with *DDC2* are likely to be involved in genome maintenance.

Among the identified genes identified, we characterized *VID22* (*YLR373C*). Originally reported to have a vesicle trafficking function (87,115), it was later found in two independent screens for genome instability causing DNA mutations (35,89) and was implicated in DNA repair (34,35). Direct observation of DNA damage response markers in a *vid22Δ* strain indicates that *VID22* plays a relevant role in protecting genomic DNA from spontaneous damage: in particular, constitutive Ddc2 and Rad53 phosphorylation (Figure 1F) in the absence of Vid22 are indicative of chronic accumulation of DNA lesions (77,116). The involvement of *VID22* in preserving genome stability is also supported by a recent genomic study (117). The continuous exposure to spontaneous DNA damage probably underlies the remarkably high frequency of gross chromosomal rearrangements observed in *vid22Δ* strains (Figure 2 and S2B). Moreover, analysis of genomic sequencing data revealed that these chromosomal aberrations are enriched in regions displaying a high propensity to form G4 structures, such as telomeres and mitochondrial DNA (Figures 3 and 4 and Supplementary Table S7). This evidence is in agreement with data indicating that Vid22 binds in the proximity of predicted G4 structures (Figure 3B, Supplementary Figure S4, Table S11 and (35)). Overall, these observations suggest a new role for Vid22 in preventing G4s from generating chromosomal aberrations.

This hypothesis is corroborated by the fact that chromosomal rearrangements at G4-prone sequences occur 15 times more frequently in the absence of *VID22* than in a wild-type background. Conversely, the GCR rate in *vid22Δ* is comparable to the wild type when the G4 motifs are absent or mutated (Figures 5B and 6F). Furthermore, loss of Vid22 sensitizes cells to the G4-stabilizing ligand TMPyP4 (Figure 5D). From the sequencing of *vid22*Δ strains genomes, we identified the *SKN7* locus on chromosome VIII as a mutational hotspots. Indeed the *SKN7* sequence is altered in 4 of 11 independent *vid22Δ* clones. The *SKN7* locus contains a G4-forming sequence that is responsible for the instability in *vid22*Δ cells. Indeed, removal of the G4 structure by point mutations suppresses the instability of the locus. Indeed, when the G4-forming sequence from *SKN7* is inserted in the *PRB1* site of the GCR reporter strain, GCRs increase nearly five-fold if *VID22* is lost, while if the G4 sequence is mutated, the GCR rate remains similar to the wild type (Figure 6F). This G4-forming sequence at *SKN7* promoter is also enriched by Vid22 ChIP and this binding is dependent on its ability to form G4, while it is independent on the presence of Tbf1-binding sites.

Interestingly, our Vid22 ChIP-seq analysis, revealed the enrichment of several additional regions that have no apparent relationship to G4s (Supplementary Figure S5), suggesting that Vid22 could bind at different genomic regions using different strategies and possibly different partners.

In the absence of *VID22*, unresolved G4-DNA structures could induce recombination intermediates, which are generally processed by specific enzymes such as Mus81 or Sgs1/Top3. Consistently, *vid22Δ* cells exhibit an increased recombination rate and are synthetic lethal/sick with *mus81Δ*, *mms4Δ* and *sgs1Δ* mutants (35,45,118). Partial or defective resolution of recombination intermediates could explain the frequent chromosomal aberrations observed in *vid22Δ* strains. Moreover, if these structures involve large chromosomal regions, their persistence could result in incorrect chromosome segregation leading to aneuploidy, which was observed in 6 of 11 *vid22Δ* strains tested (Figure 2). Curiously, we observed seven cases of whole chromosome duplication, mostly of chromosomes III, XI, and XIII. One possible explanation is that the stabilization of intermolecular-G4s between sister chromatids in the absence of *VID22* could alter chromosome segregation allowing aneuploidy. These chromosomes could have more of these structures, resulting in more susceptible alterations.

Of note, it has been shown that stabilization of G4 structures can increase the levels of DNA:RNA hybrids proximal to the G4 region, elevating genome instability (119). It is possible that, in *vid22Δ* cells, increased levels of R-loops could stimulate genomic rearrangements. However, the deletion of *VID22* does not alter the level of DNA:RNA hybrids either at the G4-prone region at the *SKN7* locus nor at the *GCN4* locus, which was reported to be enriched in DNA:RNA hybrids in several strains that accumulate stable R-loops (120) (Supplementary Figure S7). We infer from these data that the mechanism through which Vid22 protects G4-containing genomic regions from instability is not by restricting DNA:RNA hybrid accumulation.

Here, we suggest that Vid22 is able to bind G4 forming sequences both *in vitro* and *in vivo* (Figure 6D-E). Nevertheless, additional investigations are required to understand whether it contains an enzymatic activity to resolve G4 DNAs, or if it acts as a platform for specialized helicases. The existence of a genetic interactions with *SGS1* (35), which encodes a helicase capable of unwinding G4 structures, suggests that Vid22 might also contribute to G4 resolution in a distinct pathway.

The role we uncovered for *VID22* in preventing G4-dependent genome instability could have a great impact on our understanding of cancer biology. Indeed, there is a close correlation between G4 and cancer cells. Unresolved G4 structures can induce more DNA breaks and alter DNA replication, transcription and telomeric structures (121,122) severely increasing genome instability. Moreover, it is known that cancer cells present more stable G4-structures than normal cells (26). While a mammalian *VID22* orthologue has not been identified, at least eight human proteins contain a similar domain organization, that could be evolutionarily related and possess a homologous role.

## DATA AVAILABILITY

Raw sequencing data for the 11 *vid22* mutants and one control *S. cerevisiae* BY4741 strains has been deposited in the Short Read Archive (SRA) under Bioproject PRJNA646604. Data can be accessed through the following link: https://www.ncbi.nlm.nih.gov/sra/PRJNA646604.

The Vid22 ChIP-seq dataset analyzed during the current study has been deposited at the Gene Expression Omnibus (GEO) under the accession number GSE174688. Data can be accessed through the following link https://www.ncbi.nlm.nih.gov/geo/query/acc.cgi?acc=GSE174688.

All the data, including different annotations of G4 elements, Vid22 and Tbf1 peaks, and predictions of genomic rearrangements can be visualized and queried along with the complete annotation of the sacCer3 assembly of the *S. cerevisiae* genome at https://genome.ucsc.edu/s/pantaleoM/sacCer3_MC_NAR.

To facilitate the navigation through this large amount of data, we also report a series of genomic coordinates/loci which provide sensible examples of the main findings of this work:

chrVIII:511 823–515 376

chrII:4877–11 025

chrII:756 777–762 925

chrIV:1 523 402–1 525 800

chrVIII:561 295–562 643

chrXII:4927–8975

chrXII:450 697–468 827

chrXIV:6486–6948

chrXIV:782 969–784 208

## Supplementary Material

gkab1156_Supplemental_Files

## Notes

Present address: Daniele Novarina, European Research Institute for the Biology of Ageing, University of Groningen, University Medical Center Groningen, Groningen, The Netherlands.

Present address: Alessandro M. Mozzarelli, Humanitas University, Department of Biomedical Sciences, Pieve Emanuele, MI, Italy.

Present address: Anastasia Baryshnikova, Calico Life Sciences LLC, South San Francisco, CA, USA.
