# Supplementary material for: VID22 counteracts G-quadruplex-induced genome instability
Source: Nucleic Acids Res. 2021 Dec 6;49(22):12785–804. doi: 10.1093/nar/gkab1156 (PMC8682794; doi:10.1093/nar/gkab1156)
Supplement: gkab1156_Supplemental_Files [file gkab1156_supplemental_files.zip › SupplementaryDATA corrected.pdf]

SUPPLEMENTARY DATA

Supplementary Table S1. List of the yeast strains and plasmids used in the present work.

Supplementary Table S2. Colony size and SGA scores from the DDC2 overexpression screens.

Supplementary Table S3. GC composition normalization. This table reports the scaling factors applied for the normalization of depth of coverage values based on GC composition. Bins of %GC composition are indicated in the first column. Corresponding normalization factors are reported in a separate column for every strain

Supplementary Table S4. Estimates of levels of sensitivity of CNV detection. For strains showing a complete chromosomal duplication, levels of sensitivity of our in silico CNV detection assay were estimated as the ratio between the total number of overlapped genomic windows associated to duplicated chromosomes and the total number of windows showing a significantly altered coverage according to our statistical test. Strains are indicated in the first column. Estimates of sensitivity before and after the application of the GC normalization correction are reported on the second column and third column respectively.

Supplementary Table S5. DNA sequences used in Figure 6B-C-E and in Figure S6D.

Supplementary Table S6. Kinetic and equilibrium coefficients for BG4 and Vid22 binding to various probe strands. kon, koff, and Kd: values extracted from data in Figure 6B-E and S6D

Supplementary Table S7. Genomic coordinates of copy number variants (CNVs) detected in the 11 *vid22*Δ yeast strains. The table contains the complete list of predicted copy number alterations in the 11 *vid22*Δ strains analyzed in the present study. Genomic coordinates in the form of chromosome name, start coordinate and end coordinate, are reported in columns 1 to 3. Column 4 indicates the number of predicted CNVs associated with each genomic interval. Column 5 reports a comma separated list of predicted CNVs. Each CNV is indicated according to the following format <Chr Name>_<start coordinate>_<End_coordinate>_<CNV_type>_ <strain>. For CNV types "d" indicates decrease (depletion) and "D" increase (duplication).

Supplementary Table S8. Single nucleotide variants and small indels in BY and *vid22* mutants. Total number of single nucleotide substitutions and small indels (less than 5 bp in size) are reported for the wild type BY4741 yeast strain, and for each of the 11 *vid22*Δ mutants used in this study (columns). For each strain counts of indels and single nucleotide substitutions are reported on the rows. Cumulative numbers are reported in the penultimate row (Tot). The last row (Wilcoxon P) indicates the p-value, according to a Wilcoxon sum and rank test, for the over-representation of small indels and SNVs in *vid22* mutants compared to the wild type strain.

Supplementary Table S9. Enrichment of G4 elements at predicted structural genomics rearrangements. p-values according to the hyper-geometric distribution. Fold changes of

enrichment are computed by comparison with a matched number of simulated random genomic intervals of the same size. The following datasets were used for the prediction/annotation of G4 elements in the yeast genome: Capra et al. predictions according to Capra et al. (17). PDS_Marsico_et_al, and noPDS_Marsico_et_al. G4 as established by G4-seq in Marsico et al 2019 (96) with and without pyridostatin (PDS) respectively. G4_AllQuads_Kudlicki et al. G4 as predicted by the AllQuads method, using "G3 N1-7 G3 N1-7 G3 N1-7 G3 " as definition of the intra-G4 motif (97). Capra et al. was considered since it provides a highly used resource which is based on non-stringent criteria for the prediction of G4. The predictions by ALLQuad were based on more canonical and very strict criteria, while in Marsico et al they provide experimental validation of G4 forming structures by a large-scale NGS assay and it is not purely based on in-silico predictions.

Supplementary Table S10. Complete list of the genomic regions altered in *vid22* mutants and their intersections with G4 elements. The table contains the list of predicted copy number alterations in the 11 *vid22*Δ strains as described in Table_S7 intersected with predicted G4 elements. The G4 prediction dataset were described in Table_S9

Supplementary Table S11. Detailed annotation of ChIP-seq Peaks. Chr: chromosome. Start: start coordinate. End: end coordinate. LOG10-P: -log10 peak call p-value; Annotation: peak annotation. Distance to TSS: distance to nearest TSS in base pairs. Nearest Refseq: nearest refseq transcript id; Gene name: gene name.

Figure S1. Endogenous phosphorylation of Rad53 checkpoint kinase in mutants that show genomic instability. (**A**) Protein extracts from wild type, *vid22*Δ*, slx5*Δ *or slx8*Δ cells were analysed by western blotting with anti-Rad53 antibodies. The arrow indicates the phosphorylated forms of Rad53. (**B**) The cell cycle distribution of exponentially growing cultures was determined by flow cytometry of logarithmic phase *sml1*Δ, *sml1*Δ *vid22*Δ, *sml1*Δ *mec1*Δ and *sml1*Δ *mec1*Δ *vid22*Δ cultures. *SML1* deletion is necessary to suppress *mec1*Δ cell lethality. The positions of cells with 1C and 2C DNA contents are indicated.

Figure S2. Chromosomal pattern of wild type and *vid22*Δ strains. (**A**) Chromosomes were prepared from N=10 independent wild type strains and separated by PFGE. (**B**) DNA was prepared from 13 wild type and 7 independent *vid22Δ* mutants derived from the sporulation of a heterozygous diploid *VID22/vid22*Δ and chromosomes were fractionated by pulsed-field gel electrophoresis. The positions of the 16 chromosomes are indicated. ***** indicates chromosome size changes; **d** indicates chromosome duplications.

Figure S3. Comparison of expected and observed distance of CNVs prediction from G4 elements predicted from different. dataset. Frequency distribution of observed distance of predicted CNV from G4 elements is represented in blue. The expected distance distribution, estimated by 1000 independent random resampling of a matched number of genomic windows of identical size, is represented in red. Distances in base pairs (bp) are represented on the X axis, frequencies on the Y axis. The three different dataset of G4 prediction were described in Table S9.

Figure S4. Loss of Env11 does not lead to increased GCRs at G4 loci and Vid22 HU sensitivity. (**A**) The GCR rates for wt and *env11*Δ are plotted. Each data point is from an independent fluctuation test, with n ≥ 3 for each strain. The horizontal bars indicate the mean CGR rate for each strain (N=3 independent experiments). The GCR rate is calculated with the

experiments described and represented in Fig. 5. The *PRB1* locus was replaced with the G4 cassette as described in the text. An unpaired Student's *t*-test was used to compare the means of measurements and the p-value is indicated. (**B**) Tenfold serial dilutions of exponentially growing cultures of the indicated strains were plated on YPED, YPED + 100 mM HU. Images were taken after three days incubation at 28°C.

Figure S5. Summary of ChIP-seq peaks annotation and distance from G4 predicted elements. (A) The table reports a summary of the annotation of Vid22 ChIP-seq peaks. Annotation: type of genomic element. Total size: cumulative size in the genome. Vid22 Peaks: total number of Vid22 peaks associated with each element. Simulated Peaks: equivalent information for a matched number of simulated ChIP-seq peaks (see materials and methods). Log2 Enrichment: log2 fold enrichment (real vs simulated). p-value (Fisher Test): p-value for the enrichment according to a 1 tailed Fisher exact test. G4 real: number of Vid22 peaks overlapping predicted G4 elements. G4 simulated: equivalent for simulated peaks. Log2 G4 enrichment: log2 fold enrichment for proximity to G4 (real vs simulated), p-value G4 (Fisher Test): 1 tailed Fisher exact test for enrichment. (**B**) For every peak the distance to the closest element is reported. Dark purple: observed distribution. Light purple: expected distribution as established by analysis of a matched number of random genomic intervals of equivalent size. Distances, summarized in bins of 500 bps are reported on the X axis. Frequencies on the Y axis. (**C**) Zoom at a closer resolution. Only distances within 1000 bp from predicted G4 are displayed. Bins of 100 bps are used.

Figure S6. Vid22 binding to G4-DNA is independent of Tbf1 binding site, Sgs1 and its BED-domain. (**A**) List of DNA sequences of wild type and mutated Chr VIII *SKN7* locus; modified nucleotides are indicated in red; TAGGG represents the predicted Tbf1 binding site (TBS). (**B**) ChIP-qPCR of Vid22 at Chr VIII-G4 mutated in Tbf1 binding site. ChIP was performed in wild type (No Tag), Vid22-13Myc carrying G4 wild type sequence and Vid22-13Myc harbouring G4-mutated sequence; all strains used have both Tbf1-binding sites mutated as indicated in panel A. Fold enrichment of Vid22 at Chr VIII-G4 was calculated relative to the internal standard *HHT2*. Data are represented as mean ± SEM of N=3 independent experiments and the p-value is indicated. (**C**) ChIP-qPCR of Vid22 at Chr VIII-G4in presence or absence of Sgs1. Fold enrichment of Vid22 at Chr VIII-G4 was calculated as described in panel B. (**D**) Analysis of interaction of Vid22 BED-domain mutated protein with DNA forming G4 structures using the Reflective Phantom Interface (RPI) as reported in Figure 6B.

Figure S7. Vid22 loss does not lead to altered levels of DNA:RNA hybrids at the *GCN4* or *SKN7* loci. DNA:RNA hybrid immunoprecipitation (DRIP) with the S9.6 antibody in asynchronous culture of the wild-type, *vid22Δ* and *pob3-7* strains. Samples were treated (+) or not (−) in vitro with RNase HI (RNH) prior to the immunoprecipitation. Relative units are the ratio between *vid22Δ or pob3-7* and wild type percentage of input; N=3. The means and SEM of N=3 are plotted in both panels. An unpaired Student's *t*-test was used to compare the means of measurements and the p-value is indicated.

FIGURE S1

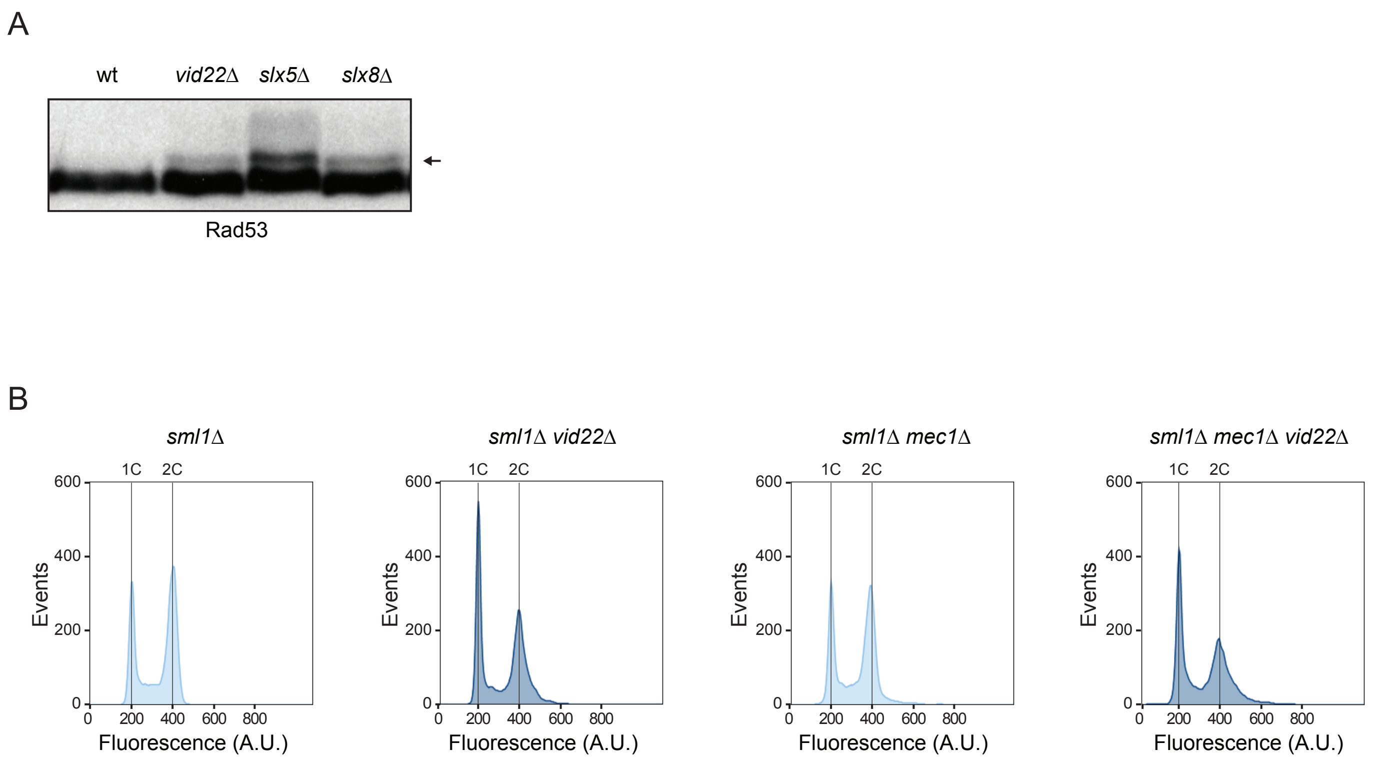

Figure S1. Endogenous phosphorylation of Rad53 checkpoint kinase in mutants that show genomic instability. (A) Protein extracts from wild type, *vid22Δ, slx5Δ or slx8Δ* cells were analysed by western blotting with anti-Rad53 antibodies. The arrow indicates the phosphorylated forms of Rad53. (B) The cell cycle distribution of exponentially growing cultures was determined by flow cytometry of logarithmic phase *sml1Δ*, *sml1Δ vid22Δ*, *sml1Δ mec1Δ* and *sml1Δ mec1Δ vid22Δ* cultures. *SML1* deletion is necessary to suppress *mec1Δ* cell lethality.
The positions of cells with 1C and 2C DNA contents are indicated.

FIGURE S2

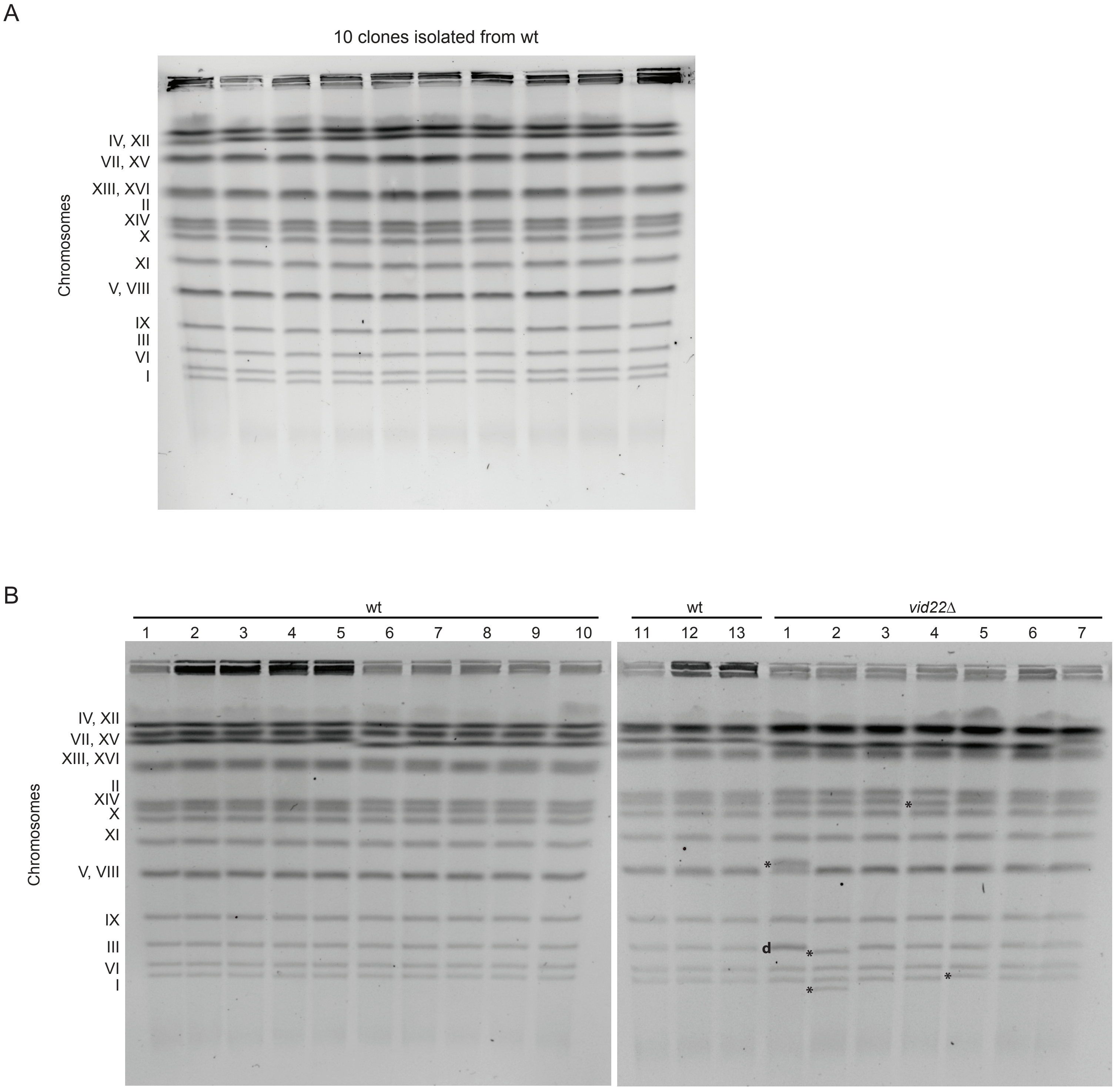

Figure S2. Chromosomal pattern of wild type and *vid22*Δ strains. (**A**) Chromosomes were prepared from N=10 independent wild-type strains and separated by PFGE. (**B**) DNA was prepared from 13 wild-type and 7 independent *vid22Δ* mutants derived from the sporulation of a heterozygous diploid *VID22/vid22*Δ and chromosomes were fractionated by pulsed-field gel electrophoresis. The positions of the 16 chromosomes are indicated. ***** indicates chromosome size changes;
d indicates chromosome duplications.

FIGURE S3

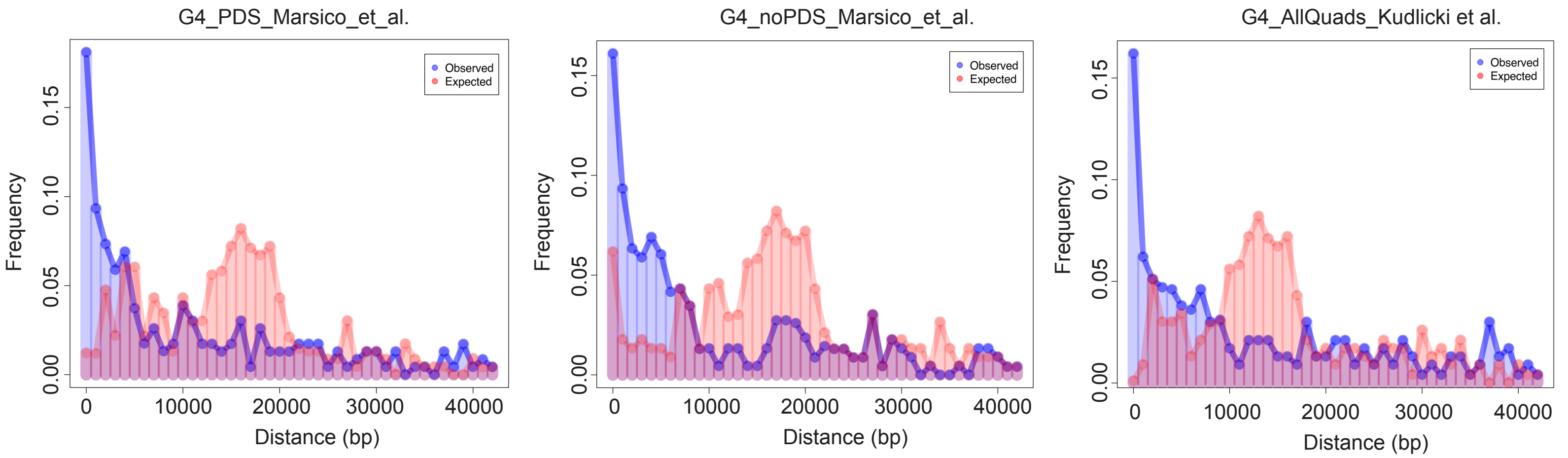

Figure S3. Comparison of expected and observed distance of CNVs prediction from G4 elements predicted from different dataset. Frequency distribution of observed distance of predicted CNV from G4 elements is represented in blue.
The expected distance distribution, estimated by 1000 independent random resampling of a matched number of genomic windows of identical size, is represented in red. Distances in base pairs (bp) are represented on the X axis, frequencies on the Y axis. The three different dataset of G4 prediction were described in Table S9.

FIGURE S4

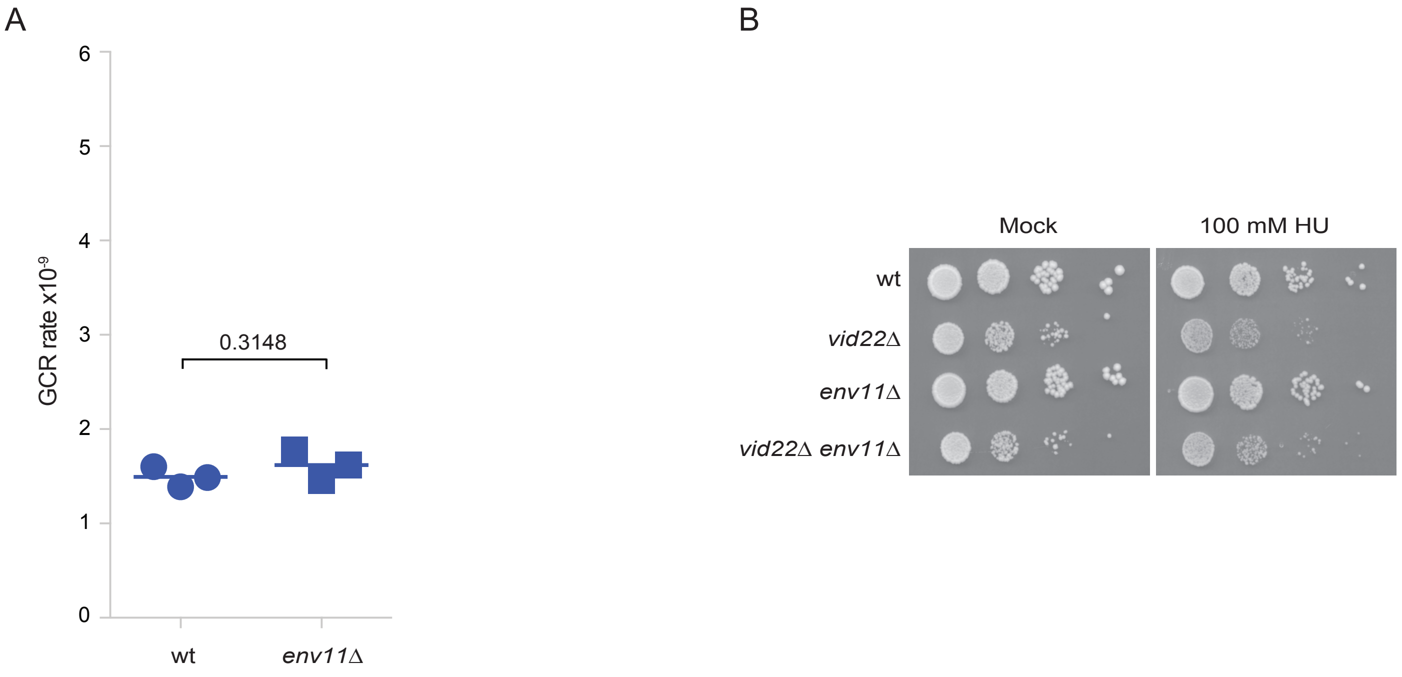

Figure S4. Loss of Env11 does not lead to increased GCRs at G4 loci and Vid22 HU sensitivity.
(**A**)The GCR rates for wt and *env11*Δ are plotted. Each data point is from an independent fluctuation test, with n ≥ 3 for each strain. The horizontal bars indicate the mean CGR rate for each strain (N=3 independent experiments). The GCR rate is calculated with the experiments described and represented in Fig. 5. The *PRB1* locus was replaced with the G4 cassette as described in the text. An unpaired Student's t-test was used to compare the means of measurements and the p-value is indicated.(**B**) Tenfold serial dilutions of exponentially growing cultures of the indicated strains were plated on YPED, YPED + 100 mM HU. Images were taken after three days incubation at 28°C.

FIGURE S5

A

Annotation	Total size (bp)	Vid22 Peaks	Simulated Peaks	Log2 Enrichment	Pvalue (Fisher Test)	G4(real)	G4 (simulated)	Log2 G4 enrichment	Pvalue (Fisher Test)
TTS	2928468	62	101	-0,7040152	9,98E-01	3	2	2,44	3,68E-01
Exon	3365496	11	125	-3,5063527	1,00E+00	1	2	5,68	1,80E-01
Intron	11604	1	0	NA	NA	0	0	NA	NA
Intergenic	2725287	108	70	0,6256045	6,03E-03	11	1	7,13	2,60E-02
Promoter	3033316	231	117	0,9813843	1,49E-07	25	2	6,33	2,27E-03
					**Total**	40	7	5,71	1,03E-06

B

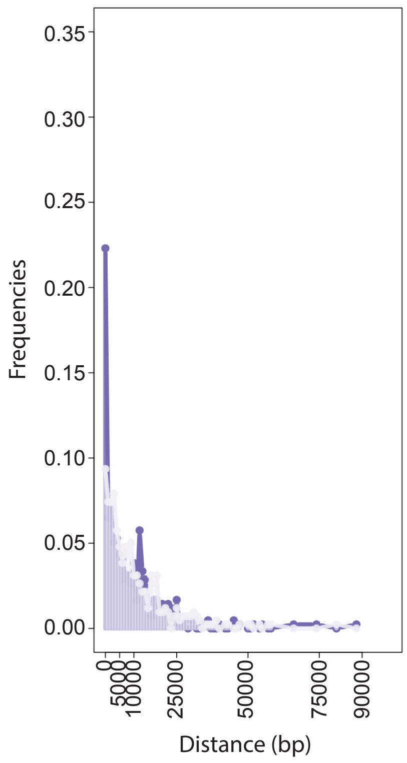

C

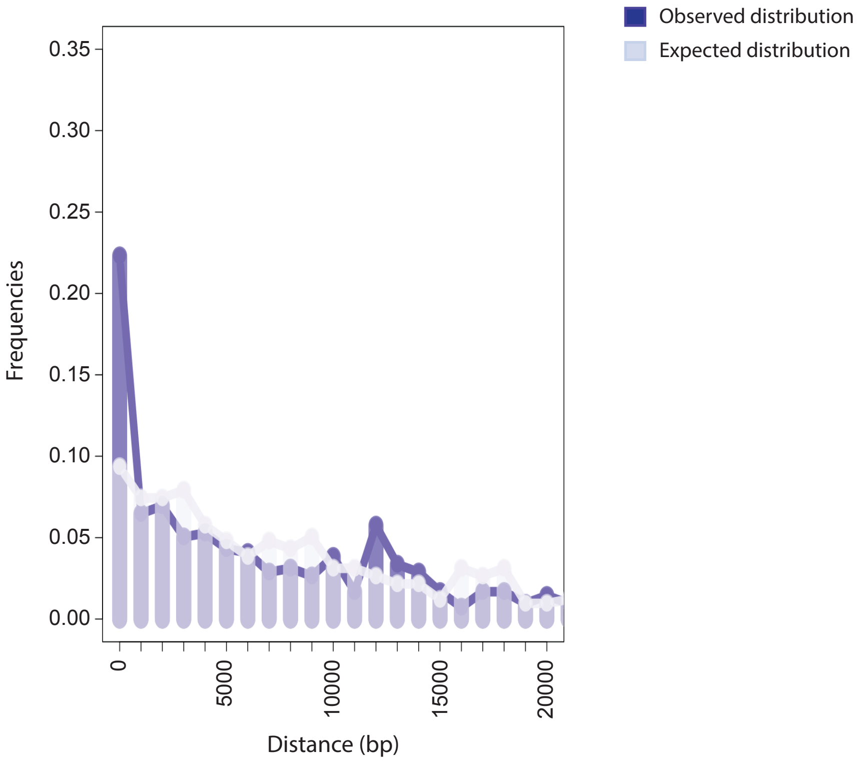

Figure S5. Summary of ChIP-seq peaks annotation and distance from G4 predicted elements.
(**A**) The table reports a summary of the annotation of Vid22 ChIP-seq peaks. Annotation: type of genomic element. Total size: cumulative size in the genome. Vid22 Peaks: total number of Vid22 peaks associated with each element. Simulated Peaks: equivalent information for a matched number of simulated ChIP-seq peaks (see materials and methods). Log2 Enrichment: log2 fold enrichment (real vs simulated). Pvalue (Fisher Test): p-value for the enrichment according to a 1 tailed Fisher exact test. G4 real: number of Vid22 peaks overlapping predicted G4 elements. G4 simulated: equivalent for simulated peaks. Log2 G4 enrichment: log2 fold enrichment for proximity to G4 (real vs simulated), Pvalue G4 (Fisher Test): 1 tailed Fisher exact test for enrichment. (**B**) For every peak the distance to the closest element is reported. Dark purple: observed distribution. Light purple: expected distribution as established by analysis of a matched number of random genomic intervals of equivalent size. Distances, summarized in bins of 500 bps are reported on the X axis. Frequencies on the Y axis. (**C**) Zoom at a closer resolution. Only distances within 20000 bp from predicted G4 are displayed.

FIGURE S6

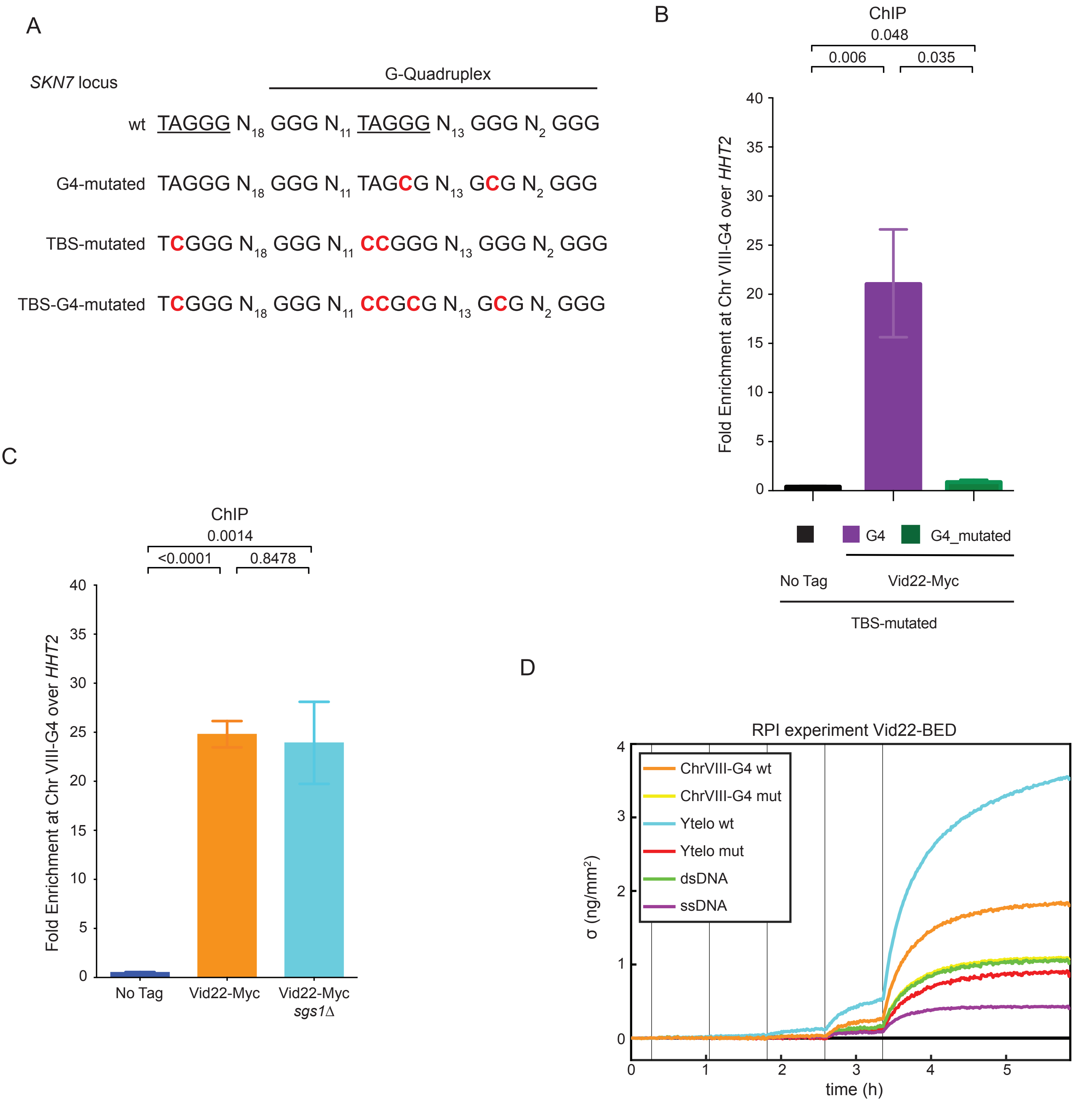

Figure S6. Vid22 binding to G4-DNA is independent of Tbf1 binding site, Sgs1 and its BED-domain. (**A**) List of DNA sequences of wild type and mutated Chr VIII *SKN7* locus; modified nucleotides are indicated in red; TAGGG represents the predicted Tbf1 binding site (TBS). (**B**) ChIP-qPCR of Vid22 at Chr VIII-G4 mutated in Tbf1 binding site. ChIP was performed in wild type (No Tag), Vid22-13Myc carrying G4 wild-type sequence and Vid22-13Myc harbouring G4-mutated sequence; all strains used have both Tbf1-binding sites mutated as indicated in panel A. Fold enrichment of Vid22 at Chr VIII-G4 was calculated relative to the internal standard *HHT2*. Data are represented as mean ± SEM of N=3 independent experiments and the p-value is indicated. (**C**) ChIP-qPCR of Vid22 at Chr VIII-G4 in presence or absence of Sgs1. Fold enrichment of Vid22 at Chr VIII-G4 was calculated as described in panel B. (**D**) Analysis of interaction of Vid22 BED-domain mutated protein with DNA forming G4 structures using the Reflective Phantom Interface (RPI) as reported in Figure 6B.

FIGURE S7

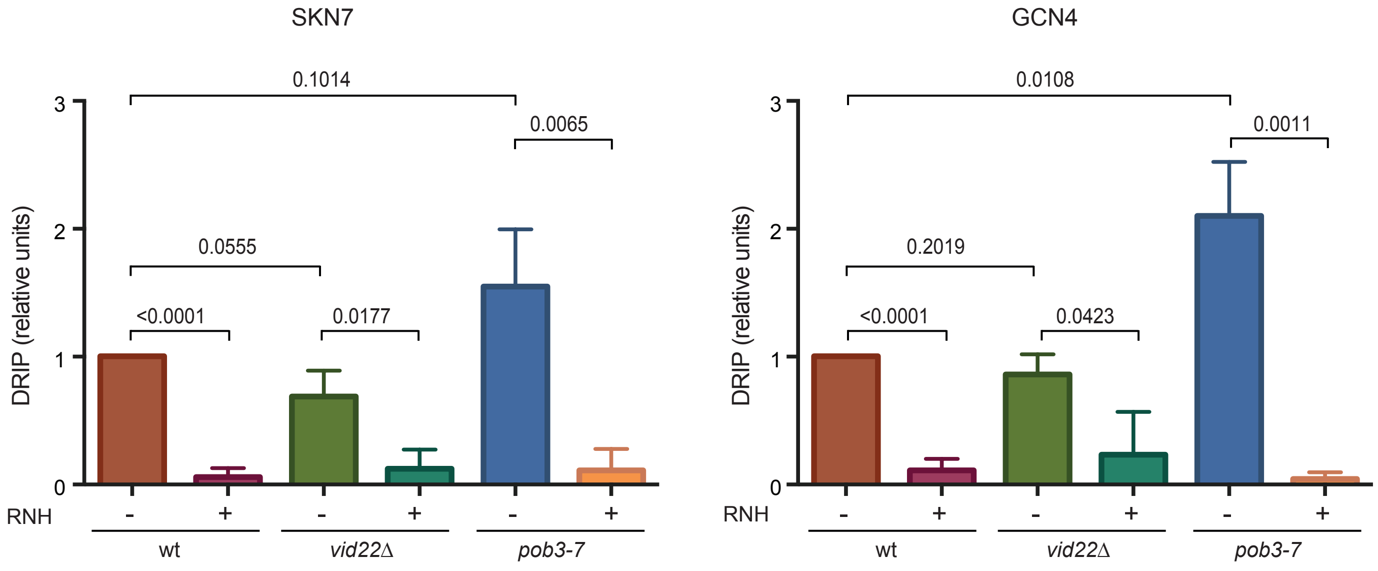

Figure S7. Vid22 loss does not lead to altered levels of DNA:RNA hybrids at the *GCN4* or *SKN7* loci. DNA:RNA hybrid immunoprecipitation (DRIP) with the S9.6 antibody in asynchronous culture of the wild type, *vid22*Δ and *pob3-7* strains. Samples were treated (+) or not (−) in vitro with RNase HI (RNH) prior to the immunoprecipitation. Relative units is the ratio between *vid22Δ* or *pob3-7* and wild type percentage of input; N=3. The means and SEM of N=3 are plotted in both panels. An unpaired Student's t-test was used to compare the means of measurements and the p-value is indicated.
